# Copper(I)–Phosphinite Complexes in Click Cycloadditions: Three‐Component Reactions and Preparation of 5‐Iodotriazoles

**DOI:** 10.1002/cctc.201600234

**Published:** 2016-06-13

**Authors:** Juana M. Pérez, Peter Crosbie, Steven Lal, Silvia Díez‐González

**Affiliations:** ^1^ Department of ChemistryImperial College LondonExhibition Road, South KensingtonLondonSW7 2AZUK; ^2^ Universidad de Alicante (Spain)

**Keywords:** azides, chemistry, copper, cycloaddition, P ligands

## Abstract

The remarkable activity displayed by copper(I)–phosphinite complexes of general formula [CuBr(L)] in two challenging cycloadditions is reported: a) the one‐pot azidonation/cycloaddition of boronic acids, NaN_3_, and terminal alkynes; b) the cycloaddition of azides and iodoalkynes. These air‐stable catalysts led to very good results in both cases and the expected triazoles could be isolated in pure form under ‘Click‐suitable’ conditions.

## Introduction

Huisgen 1,3‐dipolar cycloadditions represent a powerful methodology for the preparation of a wide range of five‐membered‐ring heterocycles.^1^, ^2^ These reactions have recently made a strong comeback owing to the huge interest attracted by the copper(I)‐catalyzed [3+2] cycloaddition of organic azides and alkynes, unarguably the best Click reaction to date. This transformation leads to the extremely efficient formation of 1,4‐disubstituted‐[1,2,3]‐triazoles as a sole regioisomer.^3^ Barely ten years after the original groundbreaking reports by Sharpless and Meldal,^4^ a myriad of ligandless as well as ligated copper systems have been reported in the literature and have found applications in diverse fields.^5^

**Scheme 1:**
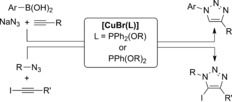
Reactions studied with the phosphi(o)nite–copper(I) complexes.

Unsurprisingly, phosphorous ligands, and PPh_3_ in particular, were among the first ligands applied to the cycloaddition of azides and alkynes.^6^, ^7^ We recently reported the excellent activity in copper‐catalyzed azide–alkyne cycloaddition (CuAAC) reactions of novel copper(I) complexes bearing phosphinite or phosphonite ligands.^8^ These complexes of general formula [CuBr(L)] are stable and can be handled with no particular precautions to exclude moisture or air. Furthermore, we showed that they outperformed related complexes with one phosphine or phosphite ligand in the synthesis of triazoles. This prompted us to further explore the potential of this family of complexes in related copper‐mediated cycloaddition reactions. Herein, we report the application of such complexes to two important reactions: the three‐component preparation of 1,4‐disubstituted triazoles from boronic acids, NaN_3_, and terminal alkynes, as well as the cycloaddition of azides and iodoalkynes (Scheme 1).

Organic azides are generally stable towards water and oxygen, and safe to use,^9^ except those of low molecular weight,^10^ which historically caused a tangible azidophobia in the chemical community.4b As a result, several approaches have been explored to avoid the handling and isolation of organic azides in [3+2] cycloadditions. These reactions are straightforward for aliphatic azides as these can be easily accessed from the corresponding halides or amines through a simple nucleophilic substitution with NaN_3_,^11^ or TfN_3_,^12^ respectively. However, reactions involving aryl azides are significantly more limited. Anilines can be reacted with *tert*‐butyl nitrite and TMSN_3_ to generate in situ the corresponding aryl azides, however, the scope of this reaction is limited.^13^ Alternatively, a proline/copper(I) system has been reported to mediate the one‐pot azidonation/cycloaddition reactions from aryl halides (iodide or bromide).^14^, ^15^

We turned our attention to the in situ preparation of aryl azides from the corresponding boronic acids because of their low toxicity and wide availability, supported by the popularity of Suzuki–Miyaura cross‐coupling reactions. To the best of our knowledge, no homogeneous, ligated copper(I) system has been reported in this context. To date, reports on this three‐component copper‐mediated transformation have focused on heterogeneous systems requiring high metal loadings (10 mol % [Cu]) and, often, a large excess of NaN_3_.^16^, ^17^

On the other hand, the preparation of 5‐iodotriazoles from the corresponding organic azides and iodoalkynes remains a challenging transformation as only a handful of efficient catalysts has been described in the literature so far.^18^, ^19^ Halogenated heterocycles are particularly interesting from a synthetic point of view, and iodotriazoles have indeed been used in several palladium‐mediated cross‐coupling reactions.^20^ Despite the clear similarities, important differences in the reactions of terminal and iodoalkynes have been reported. Whereas the ligandless combination CuSO_4_/Na ascorbate is very popular for the cycloaddition of terminal alkynes, the presence of coordinating ligands is crucial for any iodotriazole to be formed. In addition to ancillary ligands, most reported systems also require an N‐additive such as triethylamine or lutidine. These facts are aligned with the increasing evidence for different mechanistic pathways in both reactions. Fokin first suggested that cleavage of the carbon–iodine bond is not required for the cycloaddition to take place.^21^ Our DFT studies supported this and showed that either formation of a copper(III) metallacycle or direct activation of the iodoalkyne by π‐coordination of the copper catalyst accounted for the copper‐acceleration effect and regioselectivity of this cycloaddition reaction.18e

## Results and Discussion

### The three‐component reaction

In a first step, we explored the three‐component reaction between boronic acids, NaN_3_, and terminal alkynes for the one‐pot preparation of 1,4‐disubstituted triazoles. Both steps of this transformation (azidonation and cycloaddition reactions) are copper(I)‐mediated, and therefore we chose {CuBr[PPh_2_(OPh‐2‐OMe)]} **A** for the initial screening, as it had previously shown the highest activity in CuAAC reactions.^8^ With *para*‐ methoxyphenylboronic acid and phenylacetylene as model starting materials, different solvents were screened (Table 1). Reactions in acetone or acetonitrile only led to mixtures of various unknown byproducts. Reactions were cleaner in toluene but only 28 % of the expected triazole was observed as the starting boronic acid and its corresponding boroxine derivative were the major products in the crude mixture. Satisfyingly, very clean reactions were obtained in a mixture of water/MeOH (1:1). When water or MeOH were used separately the overall conversion drastically dropped owing to the presence of intermediate azide and boroxine. It is important to note that if all materials, phenylacetylene included, were added simultaneously, only 15 % conversion to the expected triazole was observed under otherwise identical reaction conditions.

**Table 1:** Solvent screening in the three‐component reaction. 
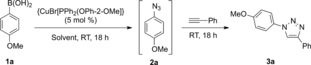

Entry^[a]^	Solvent	Ar−B(OH)_2_^[a]^ [%]	Boroxine^[a]^ [%]	Azide^[a]^ [%]	Triazole^[a]^ [%]
1	toluene	37	35	–	28
2	MeOH/water	–	–	–	>95
3	MeOH	–	18	27	54
4	water	–	22	25	53

[a] ^1^H NMR conversions are the average of at least two independent experiments.

**Scheme 2:**
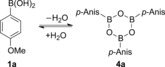
Boronic acid/boroxine equilibrium.

The trimerization of aryl boronic acids to their anhydride trimers (boroxines) is well established in the literature (Scheme 2),^22^ and it is broadly acknowledged that commercial boronic acids may contain different percentages of the corresponding boroxines. The control of this equilibrium has proven essential in several cases, either because only the boronic acids underwent the desired reaction,^23^ or because the boroxine trimer was more reactive than its monomer.^24^ Either way, we did not expect the dehydration of the boronic acid to be problematic as we ran all reactions in air and in technical solvent, or even in water.

A mild and efficient alternative to the thermal dehydration of boronic acids is the use of Lewis bases for the ligand‐facilitated trimerization of aryl boronic acids.^25^ Decoordination of the phosphinite ligand would generate a Lewis base in the reaction, however, we found no evidence for such behavior by ^1^H or ^31^P NMR spectroscopy. As only the boronic acid, and not its trimer reacted efficiently under our reaction conditions, we recrystallized and dried the starting boronic acids employed in this study whenever boroxines were observed in the starting boronic acids by ^1^H NMR spectroscopy.^26^

**Scheme 3:**
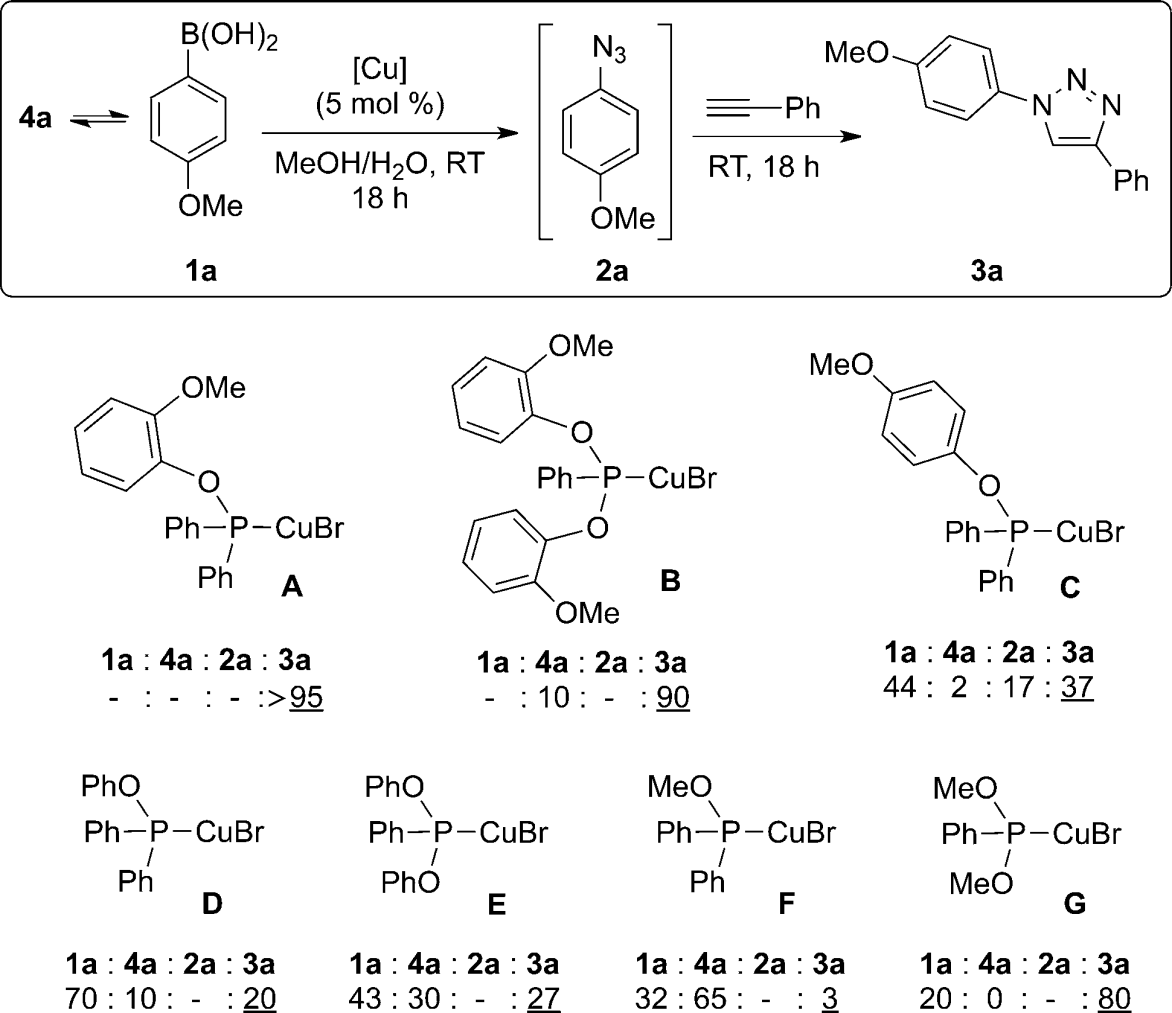
Catalyst screening in the three‐component reaction. ^1^H NMR conversions are the average of at least two independent experiments.

We next screened different phosphinite and phosphonite complexes in the model reaction (Scheme 3). With most of the catalysts tested, significant amounts of boronic acid and/or boroxine were recovered in the crude products, whereas the intermediate azide, **2 a**, was only found with catalyst **C**. In general, phosphonite complexes performed better than the corresponding phosphinite ones, with the exception of complexes **A** and **B**. Even if high conversions were obtained with phosphonite complexes **B** and **G**, **A** still outperformed all other complexes in the series.

**Scheme 4:**
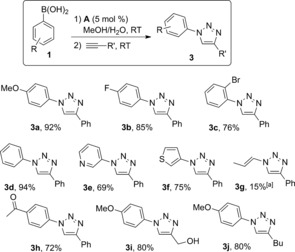
Scope of the three‐component reaction. Isolated yields are the average of at least two independent experiments. [a] Conversion determined by ^1^H NMR spectroscopy.

When the loading of **A** was halved, 40 % of the starting boronic acid was recovered, but still no azide was observed. This clearly shows that it is the azidonation, and not the cycloaddition step, that requires a higher metal loading. With an optimized catalytic system in hand, we next explored the scope of the reaction (Scheme 4). After hydrolysis and extraction with ethyl acetate, the crude solid products were washed with water, ether, and pentane to isolate the expected triazoles in an analytically pure form with no need for further purification. Different aryl boronic acids could be efficiently used in this preparation of triazoles **3**, including heteroaromatic ones. Similarly, both functionalized alkynes as well as simple aliphatic ones were successfully used under these reaction conditions.

### Cycloaddition of azides and iodoalkynes

**Scheme 5:**
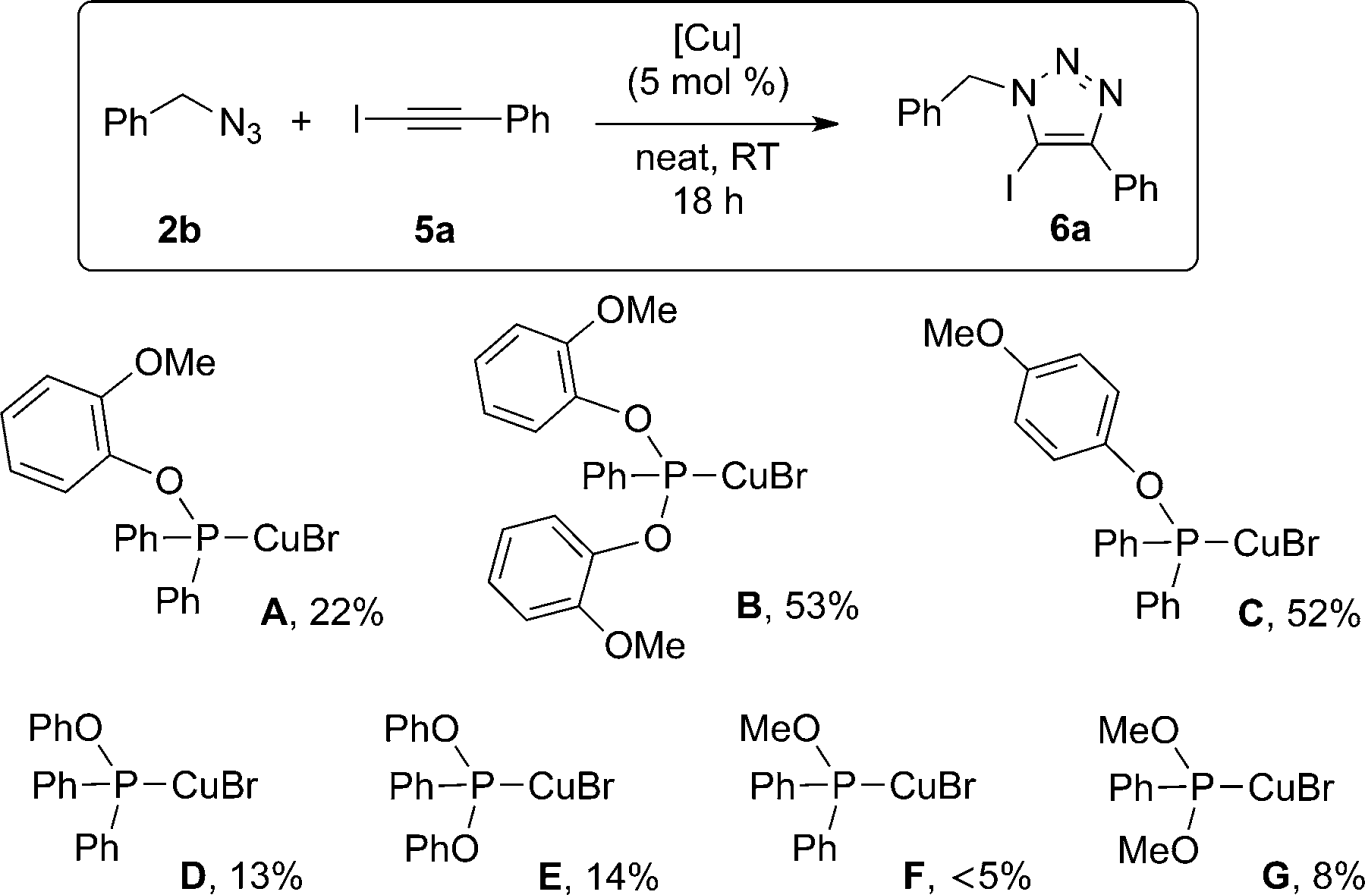
Catalyst screening in the preparation of 5‐iodotriazoles. ^1^H NMR conversions are the average of at least two independent experiments.

We started screening different phosphinite and phosphonite complexes with benzyl azide and iodoethynylbenzene as the model substrates. Reactions were carried out neat at room temperature (Scheme 5).

No reactivity trends could be drawn from the obtained results. Average conversions were observed with complexes **B** and **C**, whereas complexes with no functionalized ligands gave even poorer results. No reaction was observed in different solvents including MeCN, EtOH, toluene, and water, and only 12 % conversion was found in THF.

Several reports in the literature feature the importance of nitrogen additives in this reaction, including our own work with the related [CuI(PPh_3_)_3_] complex.18e

No improvement, however, was observed with 5 mol % of *N*,*N*‐diisopropylethylamine, 2,6‐lutidine, or triethylamine. In the presence of 1,10‐phenanthroline, the reaction conversion dropped to 25 %. Nevertheless, during the catalyst screening we noticed that complexes **B** and **C** remained active over several days^27^ and by simply raising the reaction temperature to 40 °C, full conversion into triazole **6 a** was obtained overnight with **C**. By contrast, only 20 % conversion into **6 a** was observed with **B**, which indicates that this complex is significantly more heat sensitive.

**Scheme 6:**
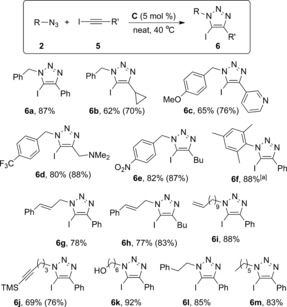
Scope of the azido–iodoalkyne cycloaddition. Isolated yields are the average of at least two independent experiments. ^1^H NMR conversions are shown in brackets for reactions that did not reach completion. [a] Reaction time=48 h.

The optimized reaction conditions were next applied to different pairs of azides and iodoalkynes (Scheme 6). In all cases, either high or complete conversions into 5‐iodotriazoles were obtained. Interestingly, whereas dehalogenated triazoles were sometimes observed in the reactions with [CuI(PPh_3_)_3_],18e this was never the case with phosphinite catalyst **C**. This allowed us to use iodoalkynes with R′=cyclopropyl, *N,N*‐dimethylaminomethyl, and butyl, which with our previous phosphine system produced ≈5–10 % of 5‐H‐triazoles (Table 2, entries 1–2). The formation of such byproducts is problematic not only because it is undesired, but also because 5‐H‐ and 5‐I‐triazoles are inseparable even by using chromatographic techniques. In this work, all formed iodotriazoles **6** could be easily isolated after a simple extraction and washing with pentane.

**Table 2:** Comparison of phosphine and phosphinite systems in the preparation of 5‐iodotriazoles. 
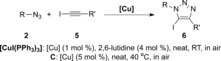

Entry	**6**	[Cu]	*t* [h]	Conv. [%]^[a]^	Yield [%]^[a]^
1	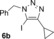	[CuI(PPh_3_)_3_]	18	74	n.d.^[b]^
		**C**	18	70	62
2	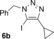	[CuI(PPh_3_)_3_]	18	79	n.d.^[b]^
		**C**	18	88	80
3	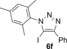	[CuI(PPh_3_)_3_]	48	24	n.d.
		**C**	48	>95	88
4	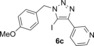	[CuI(PPh_3_)_3_]	18	<5	–
		**C**	18	76	65
5	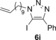	[CuI(PPh_3_)_3_]	18	<5	–
		**C**	18	>95	88
6	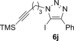	[CuI(PPh_3_)_3_]	18	<5	–
		**C**	18	76	69

[a] ^1^H NMR conversions and isolated yields are the average of at least two independent experiments. [b] 5–10 % of 5‐H‐triazole formed.

Catalyst **C** was also efficient with aryl azides, even sterically hindered ones such as mesityl azide (Table 2, entry 3). Another important advantage of this catalytic system is that it shows a wider functional group tolerance. Unsaturated C=C bonds, or pyridines did not prevent the reaction from taking place and the respective iodotriazoles could be easily isolated, whereas no reaction whatsoever had previously been observed with a phosphine catalyst (Table 2, entries 4–6). In several instances, **C** also outperformed the N‐heterocyclic carbene (NHC)‐based catalyst [CuCl(IPr)].18e Such an unequivocal change in reactivity is striking and evidence for the key role of the phosphinite ligand in the outcome of the reactions.

## Conclusions

The utility of phosphinite–copper(I) complexes in cycloaddition reactions has been explored. In particular, we have developed two competent catalytic systems for the 1,3‐dipolar cycloaddition of terminal alkynes with aryl azides generated in situ from the corresponding boronic acids as well as azides and iodoalkynes. These rely on robust air‐stable copper complexes that can be handled in air with no particular precautions. Importantly, both the reaction conditions as well as the isolation of the desired product are ‘Click‐friendly’.

Phosphinite–copper complex **A** represents the first homogenous copper(I) complex for the one‐pot azidonation/cycloaddition of boronic acids and allows the clean formation of the desired 5‐H‐triazoles with a lower metal loadings than reported for heterogeneous alternatives. On the other hand, our results in the synthesis of 5‐iodotriazoles clearly show that phosphinite–copper complex **C** is not a mere analog of [CuI(PPh_3_)_3_], with somewhat improved reactivity. Even if slightly higher metal loadings (5 instead of 1 mol %) and mild heating are required, the use of a phosphinite ligand has a profound impact on the scope of the catalytic system, both in terms of steric hindrance and functional group tolerance.

Compared with ubiquitous phosphine ligands, phosphinites (or phosphonite) ligands have only been used in a handful of copper‐catalyzed transformations.^28^ Moreover, to the best of our knowledge, no other pre‐formed copper complexes with these ligands have found applications in catalysis to date.

However, the remarkable improvement in reactivity and applicability make these system attractive candidates for other copper‐mediated processes. Efforts in this direction are currently ongoing in our laboratory and will be reported in due course.

## Experimental Section

Catalytic reactions were carried out in air and by using technical solvents without any particular precautions to exclude moisture or oxygen. The reported isolated yields for the catalytic studies are an average of two independent reactions.

**CAUTION**: Although we did not experience any problems, the cycloaddition of azides and (iodo)alkynes is highly exothermic and, as a consequence, adequate cooling should always be available when performing these reactions in the absence of solvent.

### A. Three‐component reaction (boronic acid, NaN_3_, alkyne)

In a vial fitted with a screw cap, {CuBr[PPh_2_(OPh‐2‐OMe)]} **A** (11 mg, 5 mol %), a boronic acid (0.5 mmol), sodium azide (0.5 mmol), water (1.5 mL), and MeOH (1.5 mL) were added and stirred for 18 h. Then, a terminal alkyne (0.5 mmol) was added and the solution was stirred for 18 h. The precipitate was extracted with ethyl acetate, stirred vigorously in aqueous saturated ammonium chloride solution (10 mL) for 3 h. After separation, the organic layer was concentrated under reduced pressure and the resulting solid residue was washed with water, diethyl ether and pentane, then dried under reduced pressure. In all examples, the crude products were estimated to be >95 % pure by ^1^H NMR spectroscopy.

### B. [3+2] Cycloaddition of azides and iodoalkynes

{CuBr[PPh_2_(OPh‐4‐OMe)]} **C** (11 mg, 5 mol %), azide (0.5 mmol), and iodoalkyne (0.5 mmol) were loaded into a vial fitted with a screw cap. The reaction was allowed to proceed at 40 °C for 18 h. Then, saturated aqueous ammonium chloride solution (10 mL) was added and the resulting mixture was stirred vigorously for 3 h. The resulting precipitate was filtered and washed with water and pentane, then dried under reduced pressure. In all examples, the crude products were estimated to be >95 % pure by ^1^H NMR spectroscopy.

## Supporting information

As a service to our authors and readers, this journal provides supporting information supplied by the authors. Such materials are peer reviewed and may be re‐organized for online delivery, but are not copy‐edited or typeset. Technical support issues arising from supporting information (other than missing files) should be addressed to the authors.

Supplementary
