# Supplementary material for: Copper(I)–Phosphinite Complexes in Click Cycloadditions: Three‐Component Reactions and Preparation of 5‐Iodotriazoles
Source: ChemCatChem. 2016 Jun 13;8(13):2222–6. doi: 10.1002/cctc.201600234 (PMC5089671; doi:10.1002/cctc.201600234)
Supplement: Supplementary file 1 — Supplementary [file CCTC-8-2222-s001.pdf]

Heterogeneous & Homogeneous & Bio- & Nano-
CHEMCATCHEM
CATALYSIS

Supporting Information

Copper(I)–Phosphinite Complexes in Click Cycloadditions: Three-Component Reactions and Preparation of 5-Iodotriazoles

Juana M. Pérez,[a, b] Peter Crosbie,[a] Steven Lal,[a] and Silvia Díez-González*[a]

cctc_201600234_sm_miscellaneous_information.pdf

Supporting Information – Table of Contents

1. General Considerations S2
2. Three-Component Reaction S2
3. Synthesis of Azides S5
4. Synthesis of Iodoalkynes S8
5. Preparation of 5-Iodo-[1,2,3]-Triazoles (**6**) S10
6. References S14

NMRs of New Compounds

1. GENERAL CONSIDERATIONS

All reagents were purchased from commercial sources and used without any further purification, except boronic acids. Boronic acids were recrystallized and dried whenever boraxines were observed in their ^{1}H NMR spectrum.[1] All ligands and copper catalysts were prepared following literature procedures whenever they were not commercially available.[2] Technical solvents were used in all catalytic reactions. Column chromatography and TLC were performed on silica gel (Kieselgel 60), using UV light and a phosphomolybdic acid dip to visualise the products. ^{1}H NMR (400 MHz) and ^{13}C NMR (100 MHz) spectra were recorded in $CDCl_3$ or DMSO-*d*6 on Bruker AVANCE400 spectrometers at room temperature. The chemical shifts, δ (ppm), were referenced to tetramethylsilane (^{1}H), or deuterated chloroform (^{13}C). Multiplicity is abbreviated to s (singlet), d (doublet), t (triplet), q (quartet), sxt (sextet) and m (multiplet). IR spectra were recorded with neat samples using a Perkin Elmer Spectrum 100 spectrometer fitted with an ATR accessory. High-resolution mass spectra were recorded at the Imperial College Mass Spectrometry Service Unit using ESI ionisation methods. Melting points were determined using an Electrothermal Gallenhamp apparatus fitted with a calibrated thermometer with an error of ±2°C and are uncorrected. The reported yields for the catalytic studies are an average of at least two independent reactions.

2. THREE-COMPONENT REACTION

General Procedure: In a vial fitted with a screw cap, {CuBr[PPh_2(OPh-2-OMe)]} **A** (11 mg, 5 mol %), a boronic acid (0.5 mmol), sodium azide (0.5 mmol), water (1.5 mL) and MeOH (1.5 mL) were added and stirred for 18 h. Then, a terminal alkyne (0.5 mmol) was added and the solution was stirred for 18 h. The precipitate was extracted with ethyl acetate, stirred vigorously in aqueous saturated ammonium chloride solution (10 mL) for 3 h. After separation, the organic layer was concentrated under reduced pressure and the resulting solid residue was washed with water and pentane, then dried under reduced pressure. In all examples, the crude products were estimated to be >95% pure by ^{1}H NMR.

1-(4-Methoxyphenyl)-4-phenyl-1H-[1,2,3]-triazole (3a)

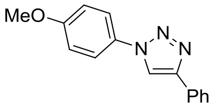

Using the general procedure from 4-methoxyphenyl boronic acid (0.076 g, 0.5 mmol) and phenlyacetylene (0.055 mL, 0.5 mmol), the title compound was isolated an off white solid (0.114 g, 92%) with spectroscopic data in accordance with the literature.[3]

^1H NMR (400 MHz, $CDCl_3$) δ 8.14 (s, 1H), 7.90 (d, *J* = 8.0 Hz, 2H), 7.69 (d, *J* = 8.0 Hz, 2H), 7.46 (t, *J* = 8.0 Hz, 3H), 7.39 (t, *J* = 8.0 Hz, 1H), 7.19 (d, *J* = 8.0 Hz, 2H), 3.88 (s, 3H); ^{13}C NMR (100 MHz, $CDCl_3$) δ 159.8, 148.2, 130.5, 130.3, 128.9, 128.3, 125.8, 122.1, 117.8, 114.8, 55.6.

1-(4-Fluorophenyl)-4-phenyl-1H-[1,2,3]-triazole (**3b**)

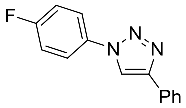

Using the general procedure from 4-fluorophenyl boronic acid (0.075 g, 0.5 mmol) and phenylacetylene (0.055 mL, 0.5 mmol), the title compound was isolated as an off white solid (0.101 g, 85%) with spectroscopic data in accordance with the literature.[4]

^1H NMR (400 MHz, $CDCl_3$) δ 8.15 (s, 1H), 7.93 (d, *J* = 7.8 Hz, 2H), 7.80–7.75 (m, 2H), 7.47 (t, *J* = 7.6 Hz, 2H), 7.38 (t, *J* = 7.6 Hz, 1H), 7.24–7.19 (m, 2H); ^{13}C NMR (100 MHz, $CDCl_3$) δ 148.3, 137.9, 135.0, 130.9 (q, *J* = 8.8 Hz), 130.2, 129.0, 128.6, 125.9, 122.5, 116.8 (q, *J* = 22.1 Hz).

1-(2-Bromophenyl)-4-phenyl-1H-[1,2,3]-triazole (**3c**)

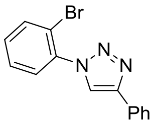

Using the general procedure from 2-bromophenyl boronic acid (0.100 g, 0.5 mmol) and phenylacetylene (0.055 mL, 0.5 mmol), the title compound was isolated as an off white solid (0.114 g, 76%) after washing with cold water and cold pentane. The obtained spectroscopic data were in accordance with the literature.[5]

^1H NMR (400 MHz, $CDCl_3$) δ 8.18 (s, 1H), 7.95–7.90 (m, 2H), 7.81–7.71 (m, 1H), 7.65–7.61 (m, 1H), 7.54–7.35 (m, 5H); ^{13}C NMR (100 MHz, $CDCl_3$) δ 147.4, 136.4, 133.8, 131.1, 130.1, 128.8, 128.45, 128.40, 128.1, 125.8, 121.6, 118.5.

1,4-Diphenyl-1H-[1,2,3]-triazole (3d)

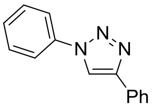

Using the general procedure from phenyl boronic acid (0.061 g, 0.5 mmol) and phenylacetylene (0.051 g, 0.5 mmol), the title compound was isolated as a white solid (0.104 g, 94%) with spectroscopic data in accordance with the literature.[3]

^1H NMR (400 MHz, $CDCl_3$) δ 8.20 (s, 1H), 7.92 (d, *J* = 7.8 Hz, 2H), 7.80 (d, *J* = 7.8 Hz, 2H), 7.59–7.53 (m, 2H), 7.50–7.43 (m, 3H), 7.41–7.35 (m, 1H); ^{13}C NMR (100 MHz, $CDCl_3$) δ 148.4, 137.1, 130.2, 129.8,128.93, 198.89, 128.8, 128.4, 125.9, 117.6.

3-(4-Phenyl-1H-[1,2,3]-triazol-1-yl)pyridine (3e)

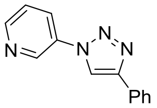

Using the general procedure from pyridin-3-yl boronic acid (0.062 g, 0.5 mmol) and phenylacetylene (0.055 mL, 0.5 mmol), the title compound was isolated as an off white solid (0.076 g, 69%) with spectroscopic data in accordance with the literature.[6]

^{1}H NMR (400 MHz, $CDCl_3$) δ 9.11–9.03 (m, 1H), 8.76–8.67 (m, 1H), 8.27 (s, 1 H), 8.23–8.18 (m, 1H), 7.92 (d, J = 7.4 Hz, 2H), 7.56–7.43 (m, 3H), 7.43–7.35 (m, 1H); ^{13}C NMR (100 MHz, $CDCl_3$) δ 149.9, 148.9, 141.4, 133.7, 129.7, 129.0, 128.7, 128.0, 125.9, 124.3, 117.4.

4-Phenyl-1-(thiophen-3-yl)-1H-[1,2,3]-triazole (3f)

Using the general procedure from thiophen-3-yl boronic acid (0.064 g, 0.5 mmol) and phenylacetylene (0.055 mL, 0.5 mmol), the title compound was isolated an off white solid (0.085 g, 75%) with spectroscopic data in accordance with the literature.[7]

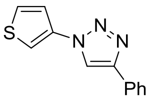

^{1}H NMR (400 MHz, $CDCl_3$) δ 8.11 (s, 1H), 7.90 (d, J = 7.2 Hz, 2H), 7.63–7.60 (m, 1H), 7.55–7.51 (m, 1H), 7.51–7.44 (m, 3H), 7.41–7.34 (m, 1H); ^{13}C NMR (100 MHz, $CDCl_3$) δ 147.9, 135.8, 130.1, 128.9, 128.4, 127.2, 125.8, 120.7, 118.0, 114.1.

1-[4-(4-Phenyl-1H-[1,2,3]-triazol-1-yl)phenyl] ethanone (3h)

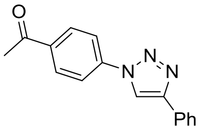

Using the general procedure from (4-acetylphenyl)boronic acid (0.082 g, 0.5 mmol) and phenylacetylene (0.055 mL, 0.5 mmol), the title compound was isolated as a yellow solid (0.95 g, 72%) with spectroscopic data in accordance with the literature.[8]

^{1}H NMR (400 MHz, DMSO-*d6*) δ 9.47 (s, 1H), 8.21 (d, J = 8.2 Hz, 2H), 8.14 (d, J = 8.2 Hz, 2H), 7.95 (d, J = 7.7 Hz, 2H), 7.55–7.48 (m, 2H), 7.41 (t, J = 7.7 Hz, 1H), 2.44 (s, 3H); ^{13}C NMR (100 MHz, DMSO-*d6*) δ 196.9, 147.6, 139.5, 136.4, 130.12, 130.06, 129.0, 128.4, 125.4, 119.7, 119.6, 26.8.

[1-(4-Methoxyphenyl)-1H-[1,2,3]-triazole-4-yl] methanol (3i)

Using the general procedure from 4-methoxyphenyl boronic acid (0.076 g, 0.5 mmol) and propargylic alcohol (0.029 mL, 0.5 mmol), the title compound was isolated a white solid (0.082 g, 80%) with spectroscopic data in accordance with the literature.[9]

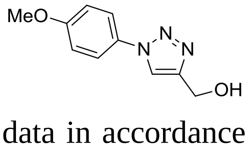

^{1}H NMR (400 MHz, DMSO-*d6*) δ 8.44 (s, 1H), 7.74 (d, *J* = 8.8 Hz, 2H), 7.11 (d, *J* = 8.8 Hz, 2H), 4.55 (s br, 2H), 3.86 (s, 3H); ^{13}C NMR (100 MHz, $CDCl_3$) δ 159.6, 148.9, 130.5, 122.0, 121.1, 115.0, 55.6, 55.1.

1-(4-Methoxyphenyl)-4-butyl-1H-[1,2,3]-triazole (3j)

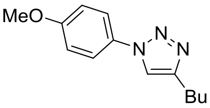

Using the general procedure from 4-methoxyphenyl boronic acid (0.076 g, 0.5 mmol) and 1-hexyne (0.057 mL, 0.5 mmol), the title compound was isolated a white solid (0.092 g, 80%) with spectroscopic data in accordance with the literature.[10]

^{1}H NMR (400 MHz, $CDCl_3$) δ 7.88 (d, *J* = 8.0 Hz, 2H), 7.88 (s, 1H), 7.06 (d, *J* = 8.0 Hz, 2H), 4.82 (s, 3H), 2.83 (t, *J* = 7.8 Hz, 2H), 1.82–1.73 (m, 2H), 1.52–1.43 (m, 2H), 1.01 (t, *J* = 7.1 Hz, 3H); ^{13}C NMR (100 MHz, $CDCl_3$) δ 159.7, 148.9, 133.7, 130.9, 122.1, 118.9, 114.8, 55.6, 31.7, 25.4, 22.5, 13.9.

3. SYNTHESIS OF AZIDES

Azides were synthesised at room temperature from the corresponding bromides or chlorides by nucleophilic substitution with sodium azide in DMSO (Alvarez procedure, Eq. 1).[11] 6-Azidohexan-1-ol (Eq. 2),[12] and 2-azido-1,3,5-trimethylbenzene[13] were prepared following previously reported procedures.

$$R^1\text{–Br} \xrightarrow[\text{DMSO, RT}]{NaN_3} R^1\text{–}N_3 \qquad (1)$$

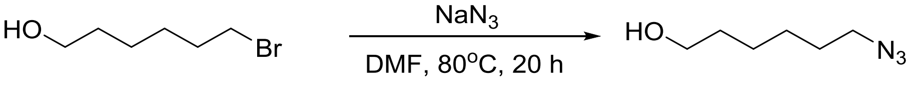

(2)

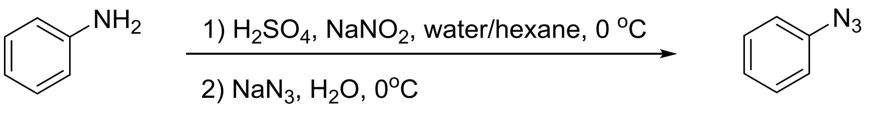

(3)

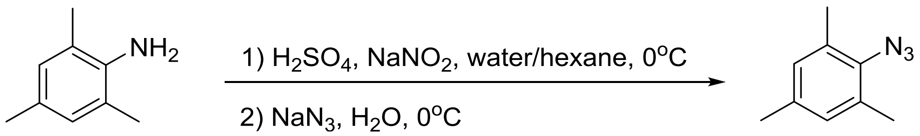

(4)

Benzyl azide (2b)

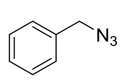

From benzyl bromide (2.40 mL, 20.0 mmol) and following the Alvarez procedure[11] (1 h, RT), the title compound was isolated as a pale yellow oil (2.60 g, 98%) with spectroscopic data in accordance with the literature.[11]

^{1}H NMR (400 MHz, $CDCl_3$) δ 7.43–7.27 (m, 5H), 4.30 (s, 2H); ^{13}C NMR (100 MHz, $CDCl_3$) δ 135.4, 128.9, 128.4, 128.3, 54.8.

1-Azidomethyl-4-methoxybenzene (2c)

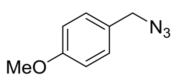

From 1-chloromethyl-4-methoxybenzene (10.85 mL, 80 mmol) and following the Alvarez procedure[11] (18 h, RT), the title compound was isolated as a pale yellow oil (12.92 g, 99%) with spectroscopic data in accordance with the literature.[14]

^{1}H NMR (400 MHz, $CDCl_3$) δ 7.25 (d, J = 8.7 Hz, 2H), 6.91 (d, J = 8.7 Hz, 2H), 4.27 (s, 2H), 3.82 (s, 3H); ^{13}C NMR (100 MHz, $CDCl_3$) δ 159.3, 129.4, 127.1, 113.8, 54.8, 53.9.

1-Azidomethyl-4-trifluoromethylbenzene (2d)

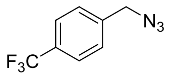

From 1-bromomethyl-4-trifluoromethylbenzene (1.03 g, 4.5 mmol) and following the Alvarez procedure[11] but using acetonitrile as the reaction solvent (20 h, RT), the title compound was isolated as a pale yellow oil (0.62 g, 73%) with spectroscopic data in accordance with the literature.[15]

^{1}H NMR (400 MHz, $CDCl_3$) δ 7.63 (d, J = 8.0 Hz, 2H), 7.44 (d, J = 8.0 Hz, 2H), 4.43 (s, 3H); ^{13}C NMR (100 MHz, $CDCl_3$) δ 139.8, 130.6 (q, J = 32.3 Hz), 128.5, 125.9 (q, J = 3.8 Hz), 123.0 (q, J = 270.0 Hz), 54.3.

1-(Azidomethyl)-4-nitrobenzene (2e)

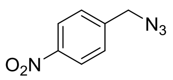

From 1-bromomethyl-4-nitrobenzene (4.32 g, 20.0 mmol) and following the Alvarez procedure[11] (4 h, RT), the title compound was isolated as a bright yellow oil (3.21 g, 90%) with spectroscopic data in accordance with the literature.[16]

^{1}H NMR (400 MHz, $CDCl_3$) δ 8.25 (d, J = 8.6 Hz, 2H), 7.51 (d, J = 8.6 Hz, 2H), 4.51 (s, 2H); ^{13}C NMR (100 MHz, $CDCl_3$) δ 147.6, 142.7, 128.6, 124.1, 53.7.

2-Azido-1,3,5-trimethylbenzene (2f)

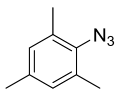

From mesityl aniline (1.82 mL, 20 mmol) and following the Fokin procedure,[13] the title compound was isolated as a yellow oil after column chromatography

(hexane, Rf = 0.71; 2.25 g, 93%). The spectroscopic data for the title compound were in accordance with the literature. [13]

^{1}H NMR (400 MHz, $CDCl_3$) δ 6.83 (s, 2H), 2.32 (s, 6H), 2.25 (s, 3H); ^{13}C NMR (100 MHz, $CDCl_3$) δ 135.3, 134.3, 131.8, 129.4, 20.7, 18.0.

(3-Azidopropenyl)benzene (2g)

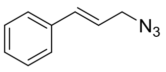

From (3-bromopropenyl)benzene (2.96 mL, 20 mmol) and following the Alvarez procedure[11] (18 h, RT), the title compound was isolated as a pale yellow oil (3.15, 99%) with spectroscopic data in accordance with the literature.[11]

^{1}H NMR (400 MHz, $CDCl_3$) δ 7.41–7.24 (m, 5H), 6.64 (d, *J* = 15.8 Hz, 1H), 6.24 (dt, *J* = 15.8; 6.6 Hz, 1H), 3.94 (d, *J* = 6.6 Hz, 2H); ^{13}C NMR (100 MHz, $CDCl_3$) δ 136.0, 134.5, 128.6, 128.2, 126.6, 122.4, 53.0.

11-Azido-1-undecene (**2h**)

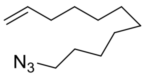

From 11-bromo-1-undecene (2.20 mL, 10 mmol) and following the Alvarez procedure[11] (18 h, RT), the title compound was isolated as a pale yellow oil (1.65 g, 85%) with spectroscopic data in accordance with the literature.[17]

^{1}H NMR (400 MHz, $CDCl_3$) δ 5.90–5.75 (m, 1H), 5.07–4.90 (m, 2H), 3.27 (t, *J* = 7.8 Hz, 2H), 2.06 (q, *J* = 7.8 Hz, 2H), 1.68–1.54 (m, 2H), 1.48–1.20 (m, 12H). ^{13}C NMR (100 MHz, $CDCl_3$) δ 140.1, 114.7, 52.0, 34.3, 29.84, 29.81, 29.55, 29.53, 29.3, 29.5, 27.2.

(5-Azido-pent-1-ynyl)trimethylsilane (2i)

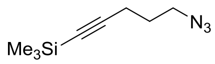

From (5-chloropent-1-ynyl)trimethylsilane (0.89 mL, 5 mmol) and following the Alvarez procedure[11] (18 h, 65°C), the title compound was isolated as a pale yellow oil (0.79 g, 87%) with spectroscopic data in accordance with the literature.[18]

^{1}H NMR (400 MHz, $CDCl_3$) δ 3.41 (t, *J* = 1.7 Hz, 2H), 2.36 (t, *J* = 1.7 Hz, 2H) 1.83–1.74 (m, 2H), 0.15 (s, 9H); ^{13}C NMR (100 MHz, $CDCl_3$) δ 105.1, 85.6, 50.0, 27.6, 17.0, -0.1.

6-Azidohexan-1-ol (2j)

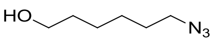

6-Bromohexan-1-ol (1.96 mL, 15 mmol) and following the Alvarez procedure[11] in DMF (18 h, 80°C), the title compound was isolated as a colorless oil (1.82 g, 85%) with spectroscopic data in accordance with the literature.[12]

^{1}H NMR (400 MHz, $CDCl_3$) δ 3.65 (t, J = 6.0 Hz, 2H), 3.27 (t, J = 6.0 Hz, 2H), 1.69–1.53 (m, 4H), 1.47–1.34 (m, 4H); ^{13}C NMR (100 MHz, $CDCl_3$) δ 62.4, 51.2, 32.3, 28.6, 26.3, 25.2.

(2-Azidoethyl)benzene (2k)

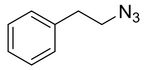

From 2-phenylethyl bromide (1.37 mL, 10.0 mmol) and following the Alvarez procedure[11] (2 h, RT), the title compound was isolated as a pale yellow oil (1.38 g, 94%) with spectroscopic data consistent with the literature.[19,16]

^{1}H NMR (400 MHz, $CDCl_3$) δ 7.36–7.17 (m, 5H), 3.49 (t, J = 7.3 Hz, 2H), 2.88 (t, J = 7.3 Hz, 2H); ^{13}C NMR (100 MHz, $CDCl_3$) δ 138.0, 128.7, 128.6, 126.7, 52.4, 35.3.

1-Azidohexane (2l)

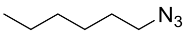

From 1-bromohexane (2.81 mL, 20 mmol) and following the Alvarez procedure[11] (18 h, RT), the title compound was isolated as a colorless oil (1.92 g, 76%) with spectroscopic data consistent with the literature.[20,21]

^{1}H NMR (400 MHz, $CDCl_3$) δ 3.26 (t, J = 7.0 Hz, 2H), 1.63–1.56 (m, 2H), 1.41–1.25 (m, 6H), 0.90 (t, J = 6.9 Hz, 3H); ^{13}C NMR (100 MHz, $CDCl_3$) δ 51.5, 31.3, 28.8, 26.4, 22.5, 14.0.

4. SYNTHESIS OF IODOALKYNES

Iodoethynylbenzene, iodoethynylcyclopropane and 1-iodo-hex-1-yne and were prepared following the procedure reported by Yamkamoto (Eq. 5).[22] 3-Iodoethynylpyridine was prepared following the procedure reported by Reddy (Eq. 6).[23] Dimethyl-(3-iodoprop-2-ynyl)amine was prepared following the procedure reported by Fokin (Eq. 7).[24]

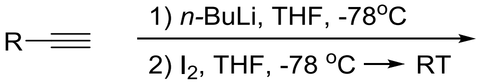

(5)

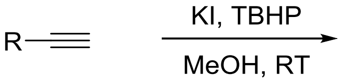

(6)

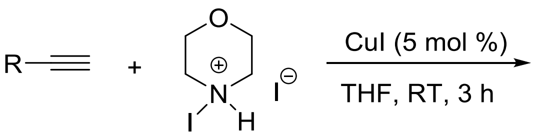

(7)

Iodoethynylbenzene (5a)

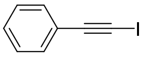

To a stirred solution of phenylacetylene (1.09 mL, 10 mmol) in THF (30 mL) *n*-BuLi (5.22 mL, 2.5 M in hexanes, 13 mmol) was added at -78°C under nitrogen atmosphere.

The resulting yellow solution was stirred for 30 min before a solution of iodine (2.67 g, 10.5 mmol) in THF (10 mL) was added. The resulting colorless solution was allowed to warm up to room temperature then poured into water (20 mL). The mixture was extracted with hexane (3 x 50 mL), and the organic phases were washed with a saturated aq. $Na_2S_2O_3$ solution (2 x 40 mL) and with brine (1 x 40 mL), dried over sodium sulfate, filtered and concentrated to give the title compound as a pale yellow oil (2.06 g, 90%) with spectroscopic data consistent with the literature.[25]

^{1}H NMR (400 MHz, $CDCl_3$) δ 7.50–7.44 (m, 2H), 7.38–7.32 (m, 3H); ^{13}C NMR (100 MHz, $CDCl_3$) δ 132.3, 128.8, 128.2, 123.4, 94.1, 6.1.

Iodoethynylcyclopropane (5b)

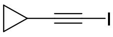
From ethynylcyclopropane (0.85 mL, 10 mmol) and following the procedure described for the preparation of **5a**, the title compound was isolated as a pale yellow oil (1.76 g, 92%) with spectroscopic data consistent with the literature.[24]

^{1}H NMR (400 MHz, $CDCl_3$) δ 1.43–1.30 (m, 1H), 0.84–0.66 (m, 4H); ^{13}C NMR (100 MHz, $CDCl_3$) δ 97.2, 9.20, 1.54, -11.8.

3-Iodoethynylpyridine (5c)

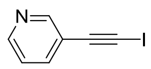
t-Butyl hydroperoxide (0.62 mL, 70% aq. solution, 4.5 mmol) was added over 30 min to a solution of 3-ethynylpyridine (0.31 g, 3 mmol) and potassium iodide (0.5 g, 3.3 mmol) in methanol (10 mL). After 18 h, the reaction was quenched with an aq. saturated $Na_2S_2O_3$ solution (10 mL), washed with brine (10 mL) and extracted with EtOAc (3 x 10 mL). The organic phases were dried over sodium sulfate, filtered and concentrated to give a crude brown oil which was purified by silica gel column chromatography (hexanes/EtOAc 2:1, Rf = 0.53) to give the title compound as a light yellow solid (0.51 g, 74%) with spectroscopic data consistent with the literature.[26]

^{1}H NMR (400 MHz, $CDCl_3$) δ 8.69 (d, *J* = 1.5 Hz, 1H), 8.54 (dd, *J* = 8.0; 4.0 Hz, 1H), 7.72 (dt, *J* = 8.0; 4.0 Hz, 1H), 7.29–7.23 (m, 1H); ^{13}C NMR (100 MHz, $CDCl_3$) δ 152.8, 148.6, 139.2, 123.2, 121.0, 90.6, 12.1.

Dimethyl(3-iodoprop-2-ynyl)amine

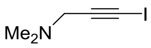
To a solution of methylprop-2-ynylamine (0.69 mL, 5.7 mmol) in THF (15 mL) were added CuI (0.048 g, 0.25 mmol) and *N*-iodomorpholine (1.88 g, 5.5 mmol). The reaction mixture was stirred at room temperature for 1 h before being filtered through a pad of neutral

alumina (THF). The organic solvent was evaporated under reduced pressure to give the title compound a light yellow solid (1.135 g, 95%) with spectroscopic data consistent with the literature.[24]

^{1}H NMR (400 MHz, $CDCl_3$) δ 3.48 (s, 2H), 2.55 (s, 6H); ^{13}C NMR (100 MHz, $CDCl_3$) δ 89.4, 50.0, 44.6, 0.0.

1-Iodohex-1-yne (5e)

From hex-1-yne (1.14 mL, 10 mmol) and following the procedure described for the preparation of **5a**, the title compound was isolated as a pale yellow oil (2.01 g, 96%) with spectroscopic data consistent with the literature.[27]

^{1}H NMR (400 MHz, $CDCl_3$) δ 2.36 (t, J = 6.8 Hz, 2H), 1.53–1.35 (m, 4H), 0.90 (t, J = 7.2 Hz, 3H); ^{13}C NMR (100 MHz, $CDCl_3$) δ 94.8, 30.6, 21.9, 20.5, 13.5, 7.7.

5. PREPARATION OF 5-IODO-[1,2,3]-TRIAZOLE6 (6)

General Procedure: In a vial fitted with a screw cap, {CuBr[PPh_2(OPh-4-OMe)]} **C** (11 mg, 5 mol %), azide (0.5 mmol) and iodoalkyne (0.5 mmol) were loaded. The reaction was allowed to proceed at 40°C for 18 h. Then, saturated aqueous ammonium chloride solution (10 mL) was added and the resulting mixture was stirred vigorously for 3 h. The resulting precipitate was filtered and washed with water and pentane, then dried under reduced pressure. In all examples, the crude products were estimated to be >95% pure by ^{1}H NMR.

1-Benzyl-5-iodo-4-phenyl-1H-[1,2,3]triazole (6a)

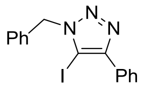
Using the general procedure from benzyl azide (0.062 mL, 0.5 mmol) and iodoethynylbenzene **5a** (0.114 g, 0.5 mmol), the title compound was isolated as an off white solid (0.157 g, 87%) with spectroscopic data consistent with the literature.[28,29]

^{1}H NMR (400 MHz, $CDCl_3$) δ 7.99–7.88 (m, 2H), 7.49–7.42 (m, 2H), 7.41–7.28 (m, 6H), 5.68 (s, 2H); ^{13}C NMR (100 MHz, $CDCl_3$) δ 150.1, 134.3, 130.1, 128.9, 128.6, 128.5, 128.4, 127.8, 127.4, 76.4, 54.3.

1-Benzyl-4-cyclopropyl-5-iodo-1H-[1,2,3]triazole (6b)

Using the general procedure from benzyl azide **2b** (0.062 mL, 0.5 mmol) and iodoethynylcyclopropane **5b** (0.096 g, 0.5 mmol), the title compound was
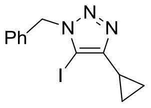

isolated as a off-white solid (0.101 g, 62%) with spectroscopic data consistent with the literature.[24]

^{1}H NMR (400 MHz, $CDCl_3$) δ 7.38–7.30 (m, 3H), 7.30–7.23 (m, 2H), 5.56 (s, 2H), 1.09–1.03 (m, 2H), 1.03–0.95 (m, 2H); ^{13}C NMR (100 MHz, $CDCl_3$) δ 153.2, 134.5, 128.9, 128.3, 127.7, 77.7, 54.0, 7.5, 7.3.

5-Iodo-1-(4-methoxybenzyl)-4-(3-pyridine)-1H-[1,2,3]triazole (6c)

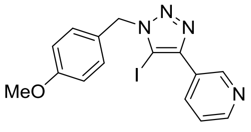

Using the general procedure from 1-azidomethyl-4-methoxybenzene **2x** (0.081 g, 0.5 mmol) and 3-iodoethynylpiridine **5c** (0.114 g, 0.5 mmol), the title complex was isolated as a yellow solid (0.127 g, 65%).

^{1}H NMR (400 MHz, $CDCl_3$) δ 9.21 (s br, 1H), 8.63 (s br, 1H), 8.27–8.20 (m, 1H), 7.42–7.35 (m, 1H), 7.33-7.27 (m, 2H), 6.92–6.85 (m, 2H), 5.61 (s, 2H), 3.78 (s, 3H); ^{13}C NMR (100 MHz, $CDCl_3$) δ 159.6, 147.9, 132.2, 131.7, 129.3, 128.5, 125.9, 121.5, 114.4, 114.1, 77.7, 55.1, 54.0. HRMS (ES): *m/z* calculated for $C_{15}H_{13}IN_4O$: 393.0212; found: 393.0229 [$(M+H)^+$].

4-Dimethylamino-5-iodo-1-(4-trifluoromethylbenzyl)-1H-[1,2,3]triazole (6d)

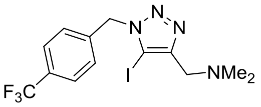

Using the general procedure from 1-azidomethyl-4-trifluoromethylbenzene **2d** (0.100 g, 0.5 mmol) and dimethyl(3-iodoprop-2-ynyl)amine **5d** (0.106 g, 0.5 mmol), the title complex was isolated as a light yellow solid (0.164 g, 80%).

^{1}H NMR (400 MHz, $CDCl_3$) δ 7.62 (d, J = 7.9 Hz, 2H), 7.36 (d, J = 7.9 Hz, 2H), 5.67 (s, 2H), 3.61 (s, 2H), 2.33 (s, 6H); ^{13}C NMR (100 MHz, $CDCl_3$) δ 148.4, 138.1, 130.7 (q, J = 32.6 Hz), 128.5 (d, J = 14 Hz), 127.9, 125.9, 81.2, 53.7, 53.5, 44.9. HRMS (ES): *m/z* calculated for $C_{13}H_{15}F_3IN_4$: 411.0293; found: 411.0304 [$(M+H)^+$].

4-*n*-Butyl-5-iodo-1-(4-nitrobenzyl)-1H-[1,2,3]triazole (6e)

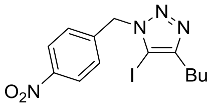

Using the general procedure from 1-(azidomethyl)-4-nitrobenzene **2e** (0.089 g, 0.5 mmol) and 1-iodohex-1-yne **5e** (0.104 g, 0.5 mmol), the title complex was isolated as a yellow solid after 24 h (0.159 g, 82%).

^{1}H NMR (400 MHz, $CDCl_3$) δ 8.23 (d, J = 8.6 Hz, 2H), 7.39 (d, J = 8.6 Hz, 2H), 5.68 (s, 2H), 2.68 (t, J = 7.7 Hz, 2H), 1.75–1.63 (m, 2H), 1.45–1.31 (m, 2H), 0.95 (t, J = 7.3 Hz, 3H); ^{13}C NMR (100 MHz, $CDCl_3$) δ 147.9, 141.4, 128.4, 124.2, 124.1, 77.2, 53.2, 30.9, 25.8, 22.2, 13.8. HRMS (ES): *m/z* calculated for $C_{13}H_{16}IN_4O_2$: 387.0318; found: 387.0318 [$(M+H)^+$].

1-(2,4,6-Trimethylphenyl)-5-iodo-4-phenyl-1H-[1,2,3]triazole (6f)

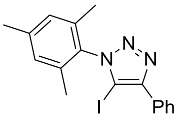

Using the general procedure from 2-azido-2,4,6-trimethylbenzene **2f** (0.161 g, 0.5 mmol) and iodoethynylbenzene **5a** (0.114 g, 0.5 mmol), the title complex was isolated as a yellow solid after 48 h (0.173 g, 88%).

^{1}H NMR (400 MHz, $CDCl_3$) δ 8.13 (d, *J* = 6.8 Hz, 2H), 7.55–7.50 (m, 2H), 7.47–7.40 (m, 1H), 7.05 (s, 2H), 2.40 (s, 3H), 1.95 (s, 6H); ^{13}C NMR (100 MHz, $CDCl_3$) δ 149.4, 140.7, 135.9, 133.0, 130.1, 129.1, 128.59, 128.56, 127.2, 79.3, 21.4, 17.4. HRMS (ES): *m/z* calculated for $C_{17}H_{17}IN_3$: 390.0467; found: 390.0473 $[(M+H)^+]$.

5-Iodo-4-phenyl-1-(3-phenylallyl)-1H-[1,2,3]triazole (6g)

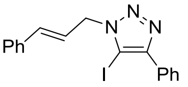

Using the general procedure from 0.074 g of **(**3-azidopropenyl)benzene **2g,** 0.114 g of iodoethynylbenzene **5a**, the title compound was isolated as a light yellow solid (0.151 g, 78%) with spectroscopic data consistent with the literature.[26]

^{1}H NMR (400 MHz, $CDCl_3$) δ 7.96 (d, *J* = 8.0 Hz, 2H), 7.50–7.44 (m, 3H), 7.42–7.36 (m, 2H), 7.34–7.27 (m, 3H), 6.67 (d, *J* = 15.8 Hz, 1H), 6.41–6.27 (m, 1H), 5.27 (dd, *J* = 6.0; 1.4 Hz, 2H); ^{13}C NMR (100 MHz, $CDCl_3$) δ 150.2, 135.6, 135.2, 132.3, 128.7, 128.6, 128.5, 128.4, 127.5, 127.4, 121.6, 76.1, 52.9.

4-*n*-Butyl-5-iodo-1-(3-phenylallyl)-1H-[1,2,3]triazole (6h)

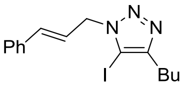

Using the general procedure from (3-azidopropenyl)benzene **2x** (0.080 g, 0.5 mmol) and 1-iodo-hex-1-yne **5e** (0.104 g, 0.5 mmol), the title complex was isolated as a yellow solid (0.283 g, 77%).

^{1}H NMR (400 MHz, $CDCl_3$) δ 7.39–7.25 (m, 5H), 6.58 (d, *J* = 16.0 Hz, 1H), 6.28 (dt, *J* = 16.0; 6.2 Hz, 1H), 5.14 (dd, *J* = 6.2; 1.5 Hz, 2H), 2.67 (t, *J* = 7.4 Hz, 2H), 1.69 (q, *J* = 7.4 Hz, 2H), 1.38 (sxt, *J* = 7.4 Hz, 2H), 0.94 (t, *J* = 7.4 Hz, 3H); ^{13}C NMR (100 MHz, $CDCl_3$) δ 152.3, 135.7, 134.9, 128.6, 128.3, 126.6, 121.9, 77.8, 52.6, 31.1, 25.8, 22.3, 13.8. HRMS (ES): *m/z* calculated for $C_{15}H_{19}IN_3$: 368.0624; found: 368.0631 $[(M+H)^+]$.

1-(Undec-11-ene)-5-iodo-4-phenyl-1H-[1,2,3]-triazole (**6i**)

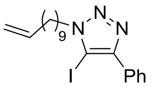

Using the general procedure from 11-azido-1-undecene **2h** (0.098 g, 0.5 mmol) and iodoethynylbenzene **5a** (0.114 g, 0.5 mmol), the title product was isolated as a light yellow solid (0.186 g, 88%).

mp 82–84 °C; ν_{max} (neat)/cm^{-1} 2917, 2850, 1641, 1411, 1344, 1227, 1153, 985, 911, 766, 694; ^{1}H NMR (400 MHz, $CDCl_3$) δ 7.95 (d, *J* = 8.0 Hz, 2H), 7.53–7.46 (m, 2H), 7.45–7.40 (m, 1H),

5.90–5.75 (m, H), 5.06–4.92 (m, 2H), 4.47 (t, *J* = 7 Hz, 2H), 2.12–1.90 (m, 4H), 1.48–1.26 (m, 12H); ^{13}C NMR (100 MHz, $CDCl_3$) δ 149.7, 139.2, 130.4, 128.6, 128.5, 127.5, 114.6, 114.2, 76.1, 51.0, 33.8, 30.0, 29.4, 29.1, 29.0, 28.9, 26.4. HRMS (ES): *m/z* calculated for $C_{19}H_{27}IN_3$: 424.1250; found: 424.1265 [$(M+H)^+$].

5-Iodo-4-phenyl-1-[5-(trimethylsilyl)-pent-4-ynyl]-1H-[1,2,3]triazole (6j)

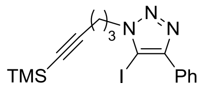

Using the general procedure from (5-azidopent-1-ynyl)trimethylsilane **2i** (0.090 g, 0.5 mmol) and (iodoethynyl)benzene **5a** (0.114 g, 0.5 mmol), the title compound was isolated as a light yellow solid (0.141 g, 69%).

mp 96–97 °C; ν_{max} (neat/cm^{-1}) 2170, 1475, 1442, 1411, 1326, 1248, 1229, 1041, 836, 757, 693, 515; ^{1}H NMR (400 MHz, $CDCl_3$) δ 7.93 (d, *J* = 8.0 Hz, 2H), 7.52–7.43 (m, 2H), 7.43–7.37 (m, 1H), 4.56 (t, *J* = 7.0 Hz, 2H), 2.39 (t, *J* = 7.0 Hz, 2H), 2.20 (q, *J* = 7.0 Hz, 2H), 0.17 (s, 9H); ^{13}C NMR (100 MHz, DMSO-*d6*) δ 148.5, 130.6, 128.3, 126.9, 106.1, 85.3, 81.4, 49.2, 28.1, 16.4; HRMS (ESI) calculated for $C_{16}H_{21}N_3ISi$ 410.0550, found 410.0550 [$(M+H)^+$].

6-(5-Iodo-4-phenyl-[1,2,3]triazol-1-yl)hexan-1-ol (6k)

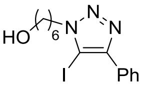

Using the general procedure from 6-azidohexan-1-ol **2j** (0.072 g, 0.5 mmol) and iodoethynylbenzene **5a** (0.114 g, 0.5 mmol), the title compound was isolated as an off-white solid (0.172 g, 92%) with spectroscopic data consistent with the literature.[26]

^{1}H NMR (400 MHz, $CDCl_3$): 7.95–7.90 (m, 2H), 7.45–7.42 (m, 2H), 7.41–7.35 (m, 1H), 4.44 (t, *J* = 7.2 Hz, 2H), 3.64 (t, *J* = 6.4 Hz, 2H), 2.02–1.86 (m, 2H), 1.64–1.56 (m, 2H), 1.52–1.33 (m, 4H); ^{13}C NMR (100 MHz, $CDCl_3$) δ 149.7, 132.0, 130.3, 128.6, 127.5, 76.3, 62.6, 50.8, 32.5, 29.9, 26.2, 25.2.

5-Iodo-1-phenethyl-4-phenyl-1H-[1,2,3]triazole (6l)

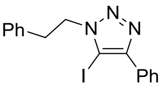

Using the general procedure from (2-azidoethyl)benzene **2k** (0.074 g, 0.5 mmol) and iodoethynylbenzene **5a** (0.114 g, 0.5 mmol), the title compound was isolated as a light yellow solid (0.159 g, 85%) with spectroscopic data consistent with the literature.[26]

^{1}H NMR (400 MHz, $CDCl_3$) δ 7.97–7.90 (m, 2H), 7.52–7.45 (m, 2H), 7.44–7.38 (m, 1H), 7.36–7.25 (m, 3H), 7.24–7.19 (m, 2H), 4.71 (t, *J* = 7.7 Hz, 2H), 3.27 (t, *J* = 7.7 Hz, 2H); ^{13}C NMR (100 MHz, DMSO-*d6*) δ 148.3, 137.1, 130.6, 128.8, 128.6, 128.4 , 128.2, 126.9, 126.7, 81.6, 51.3, 35.5.

1-Hexyl-5-iodo-4-phenyl-1H-[1,2,3]triazole (6m)

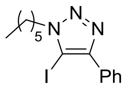

Using the general procedure from 1-azidohexane **2l** (0.074 g, 0.5 mmol) and iodoethynylbenzene **5a** (0.114 g, 0.5 mmol), the title compound was isolated as a light yellow solid (0.112 g, 83%) with spectroscopic data consistent with the literature.[26]

^{1}H NMR (400 MHz, $CDCl_3$) δ 8.00–7.88 (m, 2H), 7.51–7.43 (m, 2H), 7.95–7.90 (m, 2H), 7.43–7.35 (m, 1H), 4.45 (t, J = 7.4 Hz, 2H), 2.03–1.87 (m, 2H), 1.46–1.27 (m, 6H), 0.89 (t, J = 4.5 Hz, 3H); ^{13}C NMR (100 MHz, $CDCl_3$) δ 149.6, 132.0, 130.3, 128.5, 127.5, 76.1, 51.0, 31.2, 29.9, 26.1, 22.4, 14.0.

6. REFERENCES

[1] M. Storgaard, J. A. Ellman, *Org. Synth.* **2009**, *86*, 360–373.

[2] S. Lal, J. McNally, A. J. P. White, S. Díez-González, *Organometallics* **2011**, *30*, 6225–6232.

[3] Z.-X. Wang, H.-L. Qin, *Chem. Commun.* **2003**, 2450–2451.

[4] a) Z. Chen, Q. Yan, Z. Liu, Y. Xu, Y. Zhang, *Angew. Chem. Int. Ed.* **2013**, *52*, 13325–13328; b) H. Hu, A. Zhang, L. Ding, X. Lei, L. Zhang, *Molecules* **2008**, *13*, 556–566.

[5] a) X. Meng, X. Xu, T. Gao, B. Chen, *Eur. J. Org. Chem.* **2010**, 5409–5414 ; b) D. Wang, N. Li, M. Zhao, W. Shi, C. Ma, B. Chen, *Green Chem.* **2010**, *12*, 2120–2123.

[6] B. Chattopadhyay, C. I. R. Vera, S. Chuprakov, V. Gevorgyan, *Org. Lett.* **2010**, *12*, 2166–2169.

[7] S. Mohammed, A. K. Padala, B. A. Dar, B. Singh, B. Sreedhar, R. A. Vishwakarma, S. B. Bharate, *Tetrahedron* **2012**, *68*, 8156–8162.

[8] K. Barrat, A. D. Moorhouse, J. E. Moses, *Org. Lett.* **2007**, *9*, 1809–1811.

[9] N. Boechat, V. F. Ferreira, S. B. Ferreira, M. de L. G. Ferreira, F. de C. da Silva, M. M. Bastos, M. dos S. Costa, M. C. S. Lourenc, A. C. Pinto, A. U. Krettli, A. C. Aguiar, B. M. Teixeira, N. V. da Silva, P. R. C. Martins, F. A. F. M. Bezerra, A. L. S. Camilo, G. P. da Silva, C. C. P. Costa, *J. Med. Chem.* **2011**, *54*, 5988–5999.

[10] M. Gholinejad, N. Jeddi, *ACS Sustainable Chem. Eng.* **2014**, *2*, 2658–2665.

[11] S. G. Alvarez, M. T. Alvarez, *Synthesis* **1997**, 413–414.

[12] H. Li, A. C. Fahrenbach, A. Coskun, Z. Zhu, G. Barin, Y.-L. Zhao, Y. Y. Botros, J.-P. Sauvage, J. F. Stoddart, *Angew. Chem. Int. Ed.* **2011**, *50*, 6782–6788.

[13] S. W. Kwok, J. R. Fotsing, R. J. Fraser, V. O. Rodionov, V. V. Fokin, *Org. Lett.* **2010**, *12,* 4217–4219.

[14] U. Sirion, H. J. Kim, J. H. Lee, J. W. Seo, B. S. Lee, S. J. Lee, S. J. Oh, D. Y. Chi, *Tetrahedron Lett.* **2007**, *48*, 3953–3957.

[15] K. Asano, S. Matsubara, *Org. Lett.* **2010**, *12*, 4988–4991.

[16] S. Díez-González, A. Correa, L. Cavallo, S. P. Nolan, *Chem. Eur. J.* **2006**, *12*, 7558–7564.

[17] D. Bandyopadhyay, Y-Y. Luk and D. Prashar, *Chem. Commun*. 2011, **47**, 6165–6167.

[18] L. Díaz, J. Bujons, J. Casas, A. Llebaria, A. Delgado, *J. Med. Chem.* **2010**, *53*, 5248–5255.

[19] M. Ito, K.-i. Koyakumaru, T. Ohta, H. Takaya, *Synthesis* **1995**, 376–378.

[20] Y. Masuda, M. Hoshi, A. Arase, *Bull. Chem. Soc. Jpn.* **1984**, *57,* 1026–1030.

[21] S. Lal, S. Díez-González, *J. Org. Chem*. **2011**, *76*, 2367–2373.

[22] D. L. Usanov, H. Yamamoto, *J. Am. Chem. Soc.* **2011**, *133*, 1286–1289.

[23] K. R. Reddy, M. Venkateshwar, C. U. Maheswari, P. S. Kumar, *Tetrahedron Lett.* **2010**, *51*, 2170–2173.

[24] E. Hein, J. C. Tripp, L. B. Krasnova, K. B. Sharpless, V. V. Fokin, *Angew. Chem. Int. Ed.* **2009**, *48,* 8018–8021.

[25] H. A. Stefani, R. Cella, F. A. Dörr, C. M. P. de Pereira, F. P. Gomes, Zeni, G. *Tetrahedron Lett.* **2005**, *46*, 2001–2003.

[26] S. Lal, H. S. Rzepa, S. Díez-González, *ACS Catal.* **2014**, *4*, 2274–2287.

[27] J. Yan, J. Li, D. Cheng, *Synlett* **2007**, 2442–2444.

[28] Y.-M. Wu, J. Deng, Y. Li, Q.-Y. Chen, *Synthesis* **2005**, 1314–1318.

[29] P. Dinér, T. Andersson, J. Kjellén, K. Elbing, S. Hohmann, M. Grøtli, *New J. Chem.* **2009**, *33*, 1010–1016.

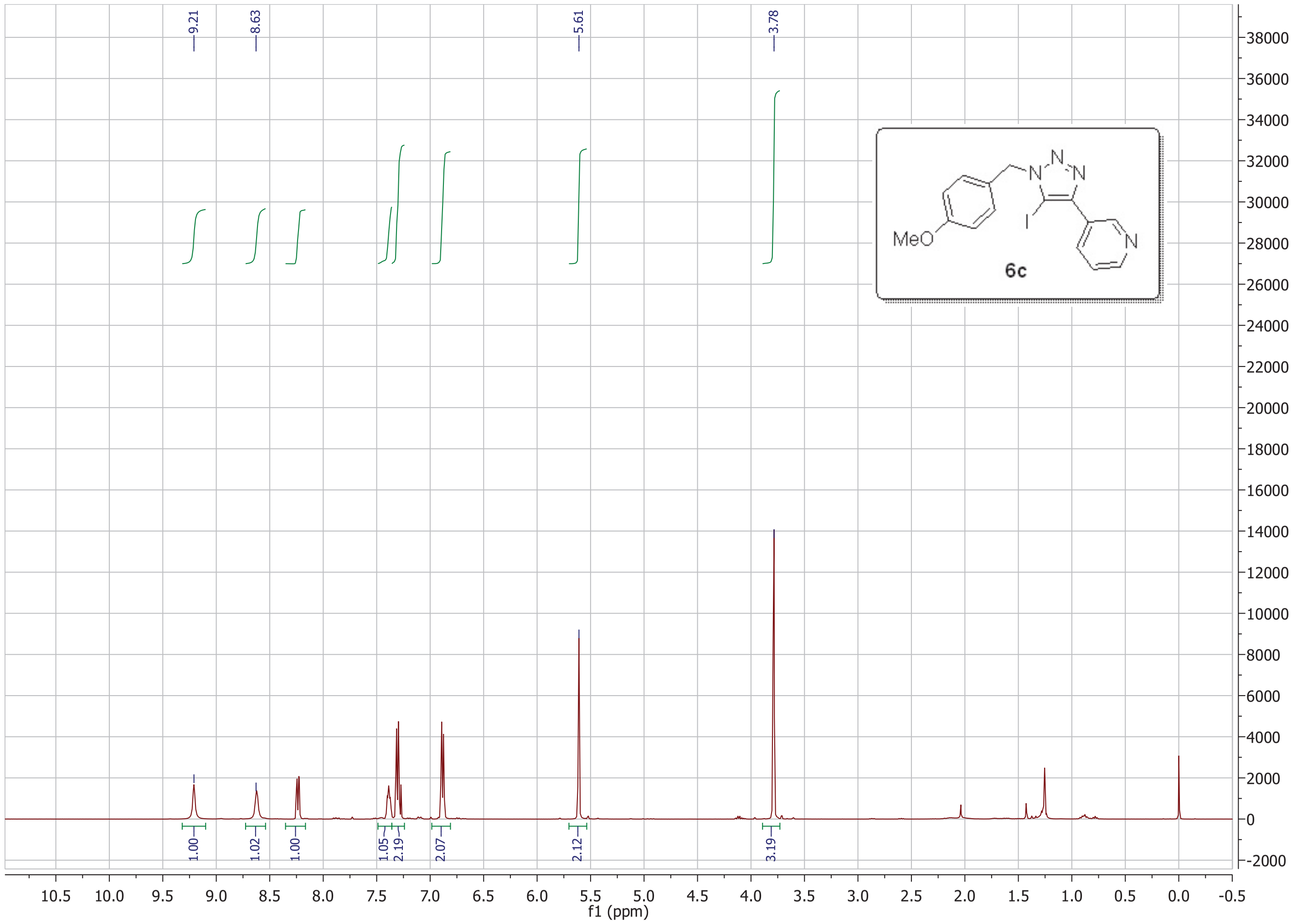

9.21
8.63
5.61
3.78
1.00
1.02
1.00
1.05
2.19
2.07
2.12
3.19
MeO
6c
f1 (ppm)

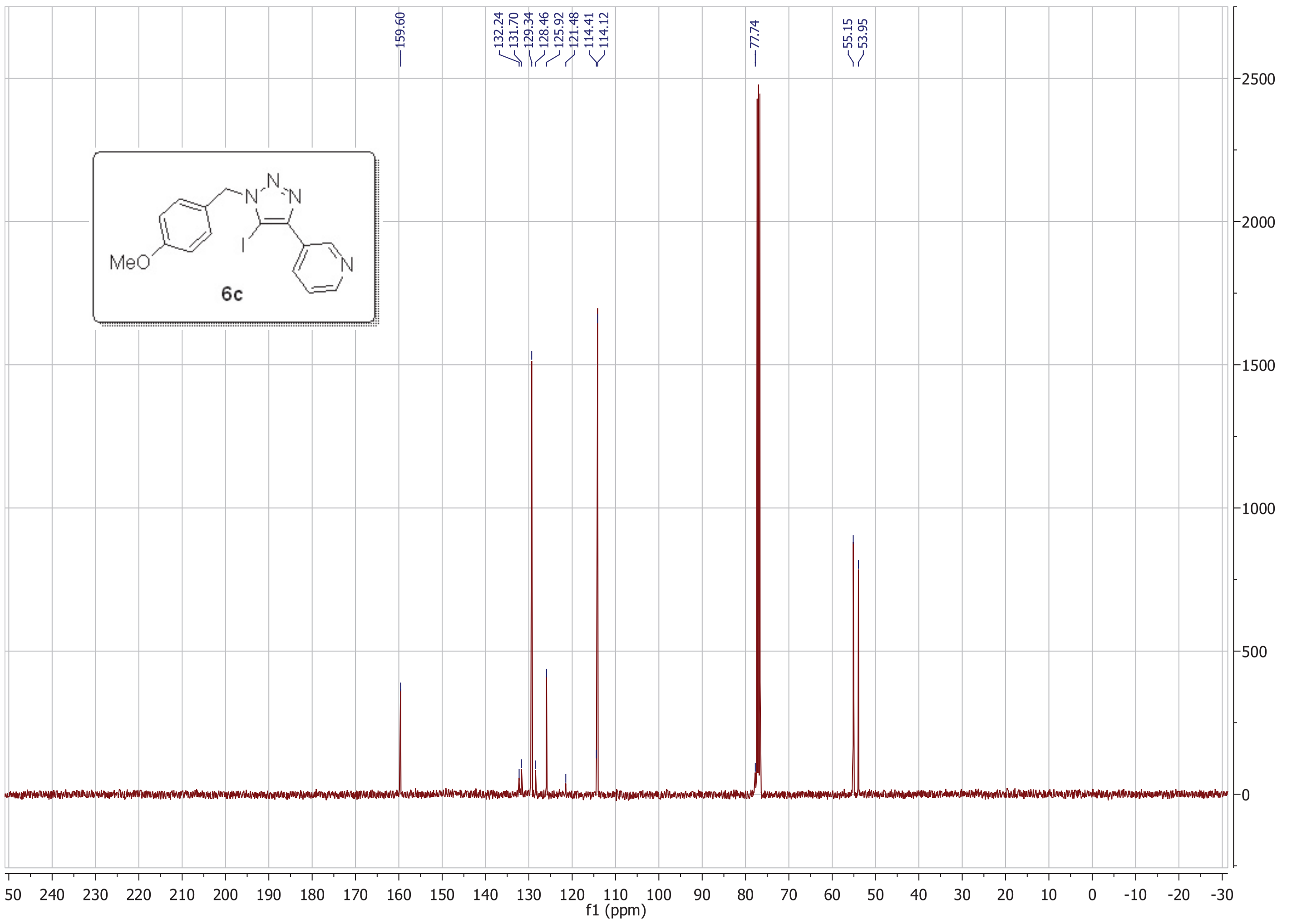

159.60
132.24
131.70
129.34
128.46
125.92
121.48
114.41
114.12
77.74
55.15
53.95
MeO
6c
f1 (ppm)

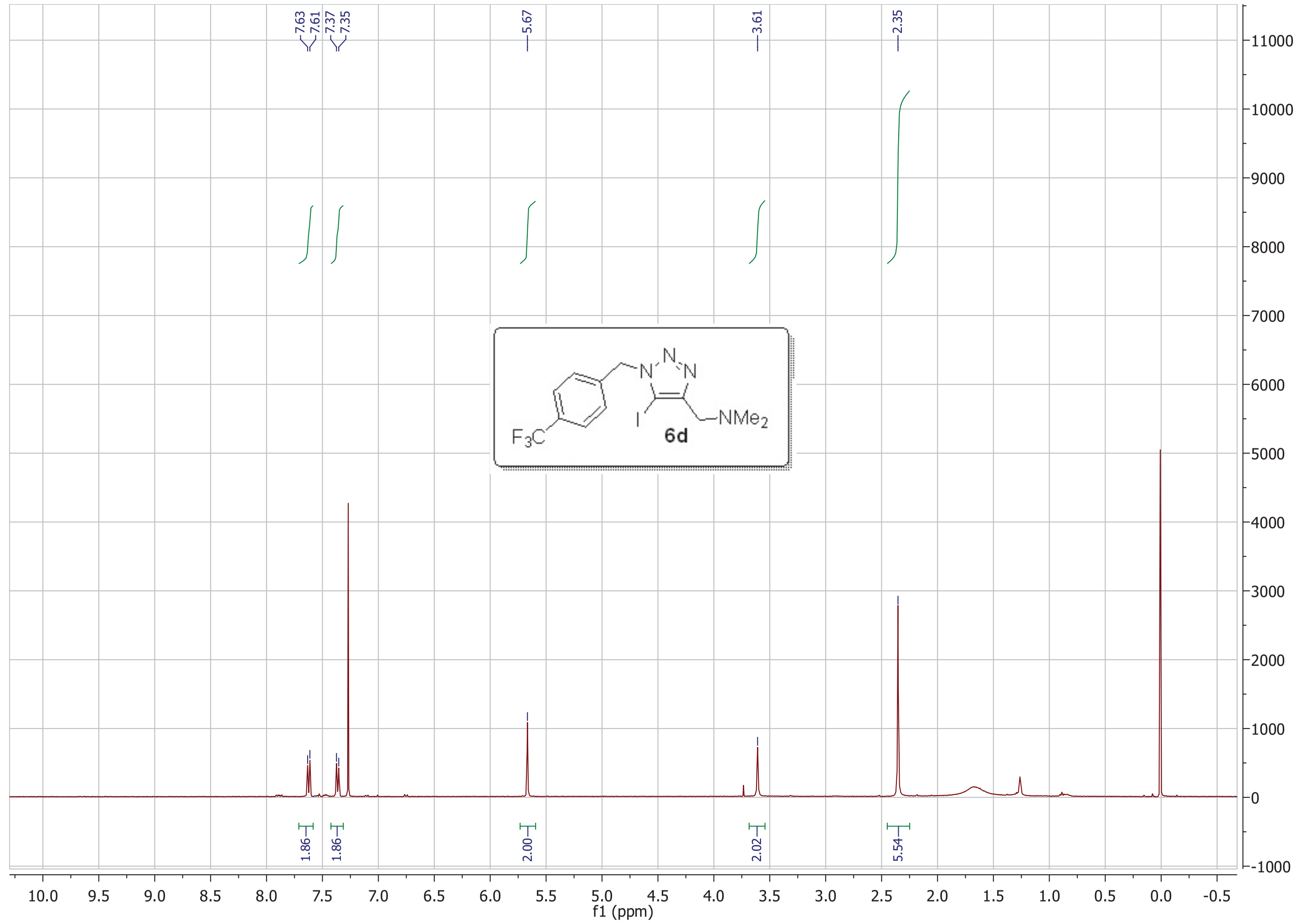
7.63
7.61
7.37
7.35
5.67
3.61
2.35
F3C
N
N
N
I
NMe2
6d
1.86
1.86
2.00
2.02
5.54
f1 (ppm)

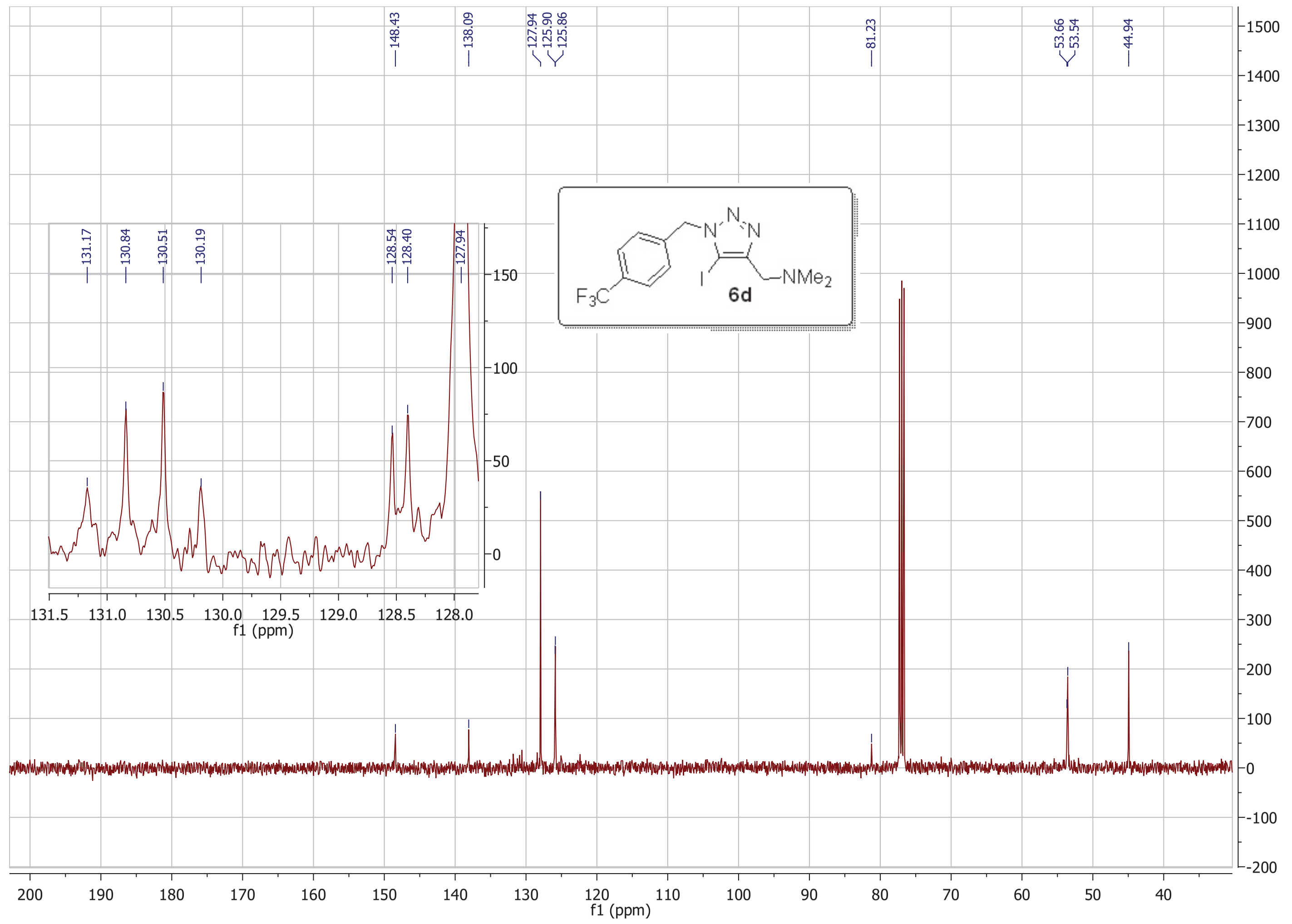
148.43
138.09
127.94
125.90
125.86
81.23
53.66
53.54
44.94
131.17
130.84
130.51
130.19
128.54
128.40
127.94
F3C
N
N
N
I
NMe2
6d
f1 (ppm)
f1 (ppm)

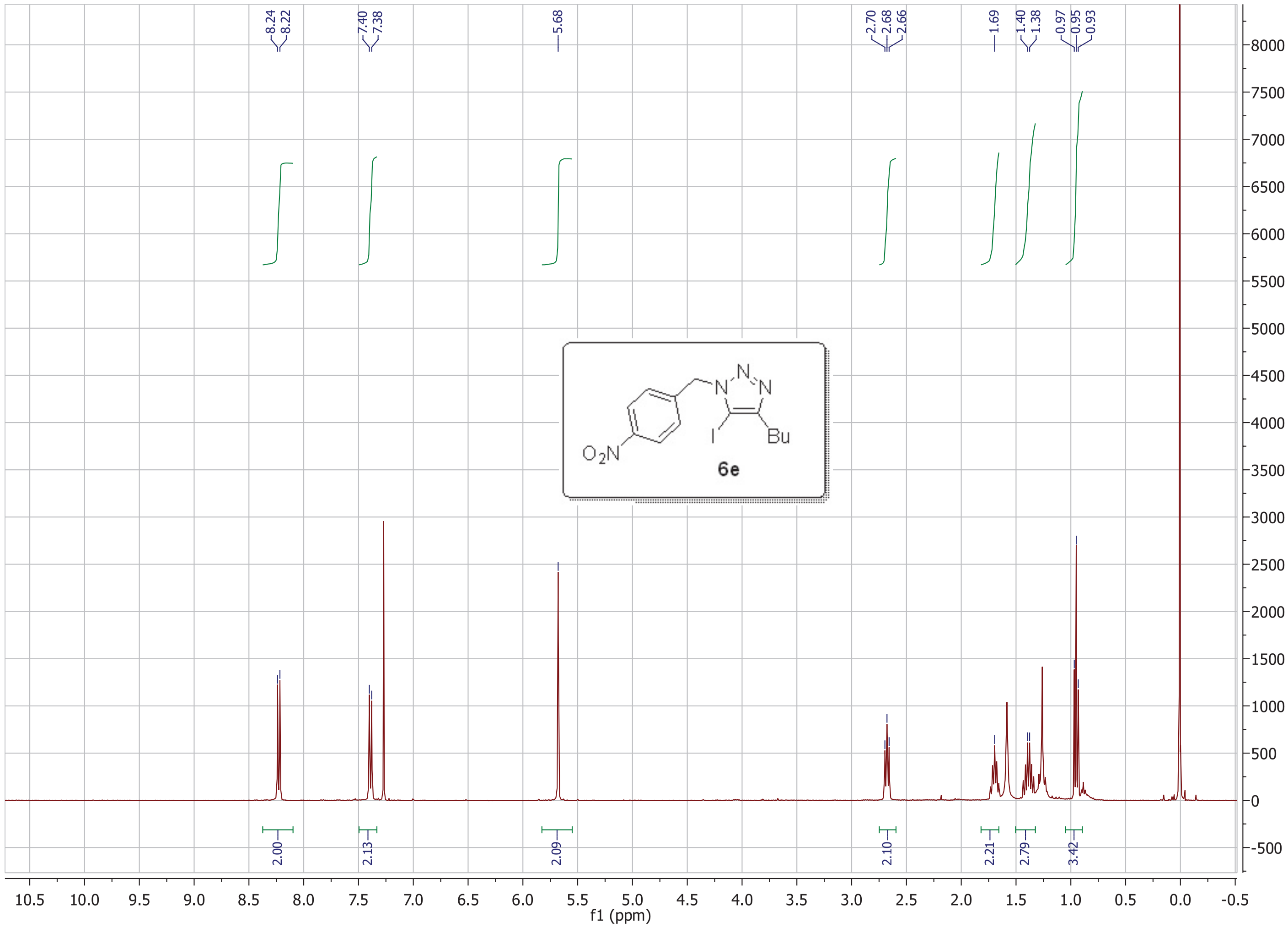
8.24
8.22
7.40
7.38
5.68
2.70
2.68
2.66
1.69
1.40
1.38
0.97
0.95
0.93
O2N
N
N
N
I
Bu
6e
2.00
2.13
2.09
2.10
2.21
2.79
3.42
f1 (ppm)

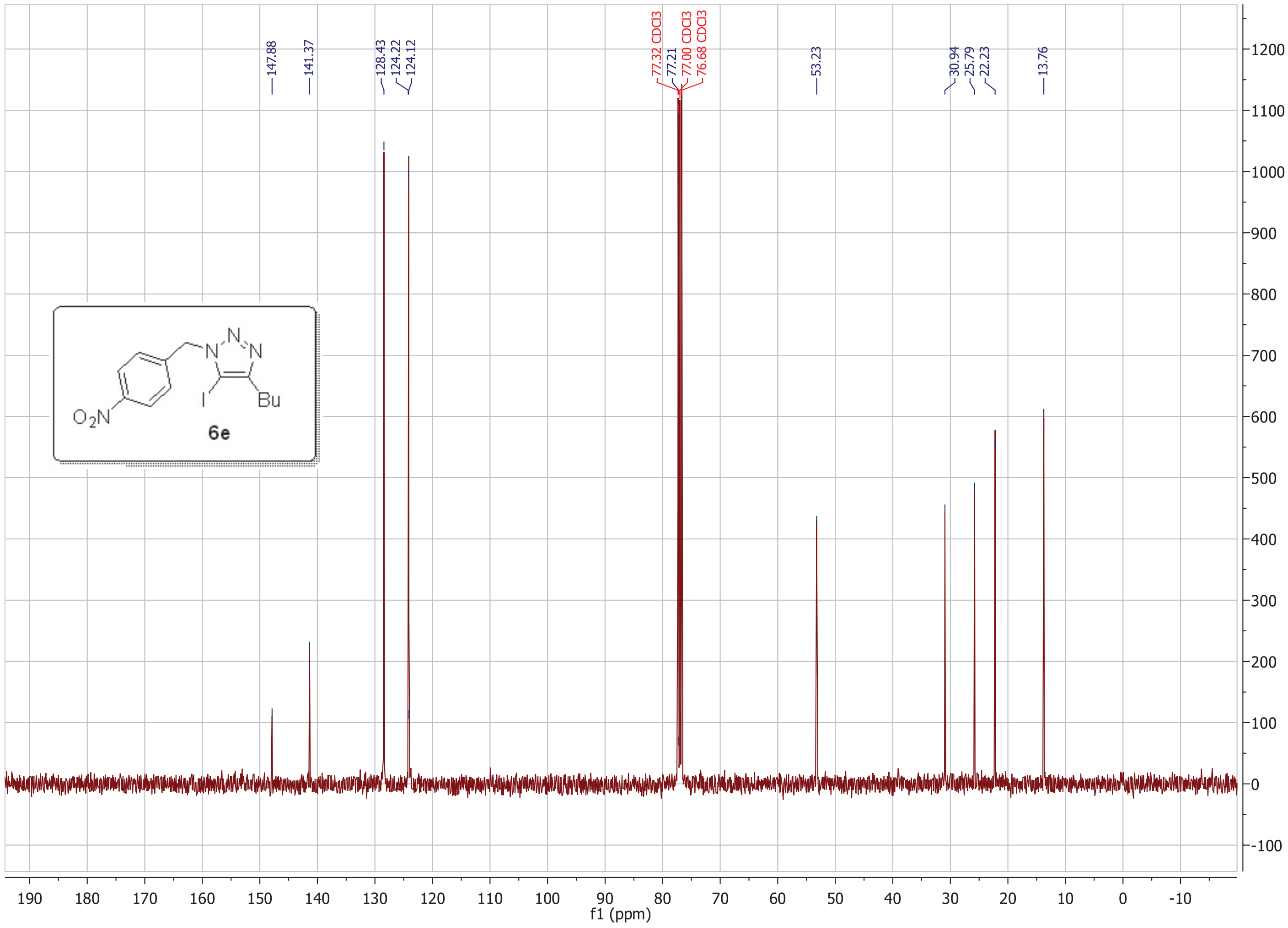
147.88
141.37
128.43
124.22
124.12
77.32 CDCl3
77.21
77.00 CDCl3
76.68 CDCl3
53.23
30.94
25.79
22.23
13.76
O2N
N
N
N
I
Bu
6e
f1 (ppm)

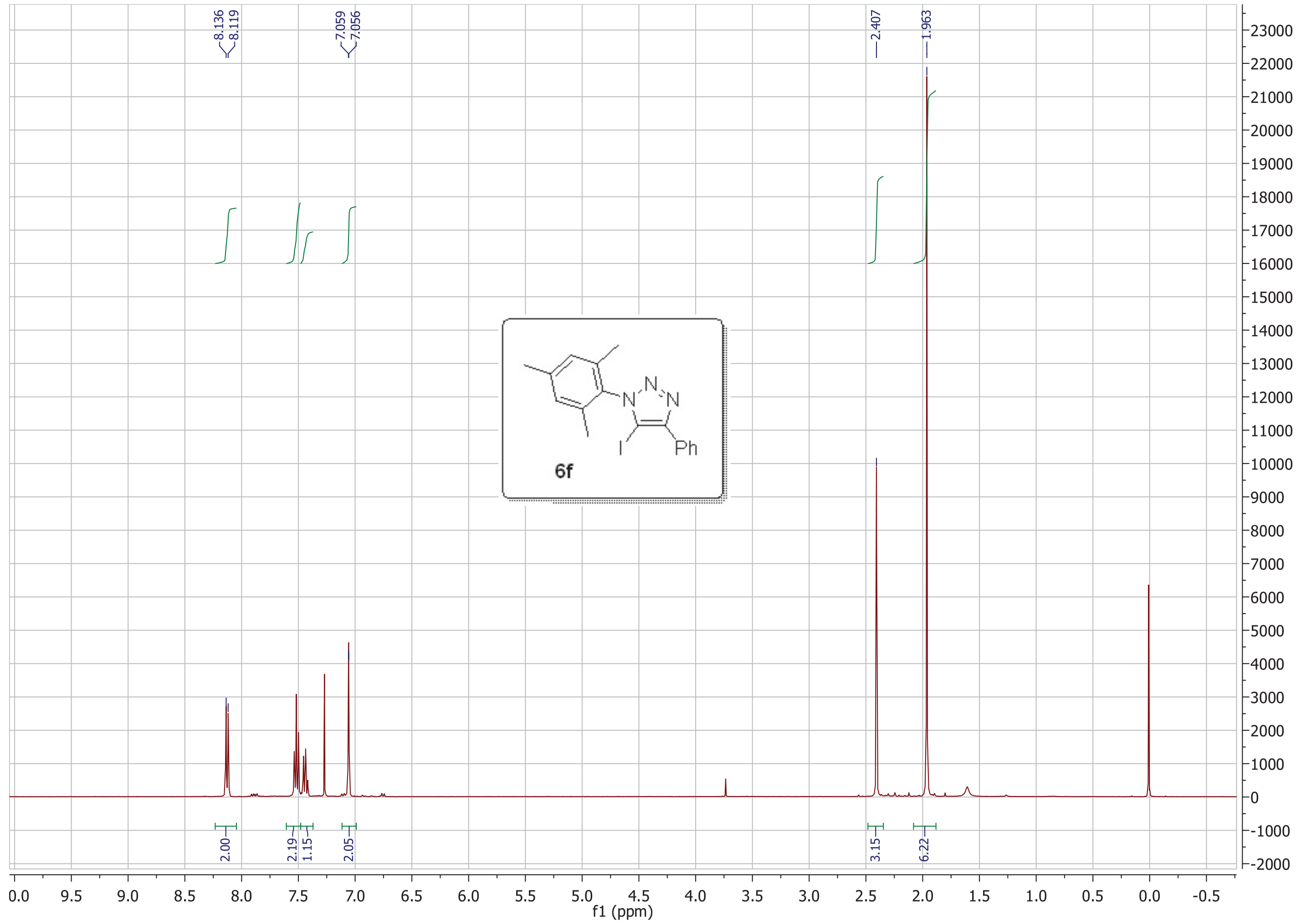
8.136
8.119
7.059
7.056
2.407
1.963
2.00
2.19
1.15
2.05
3.15
6.22
6f
Ph
I
f1 (ppm)

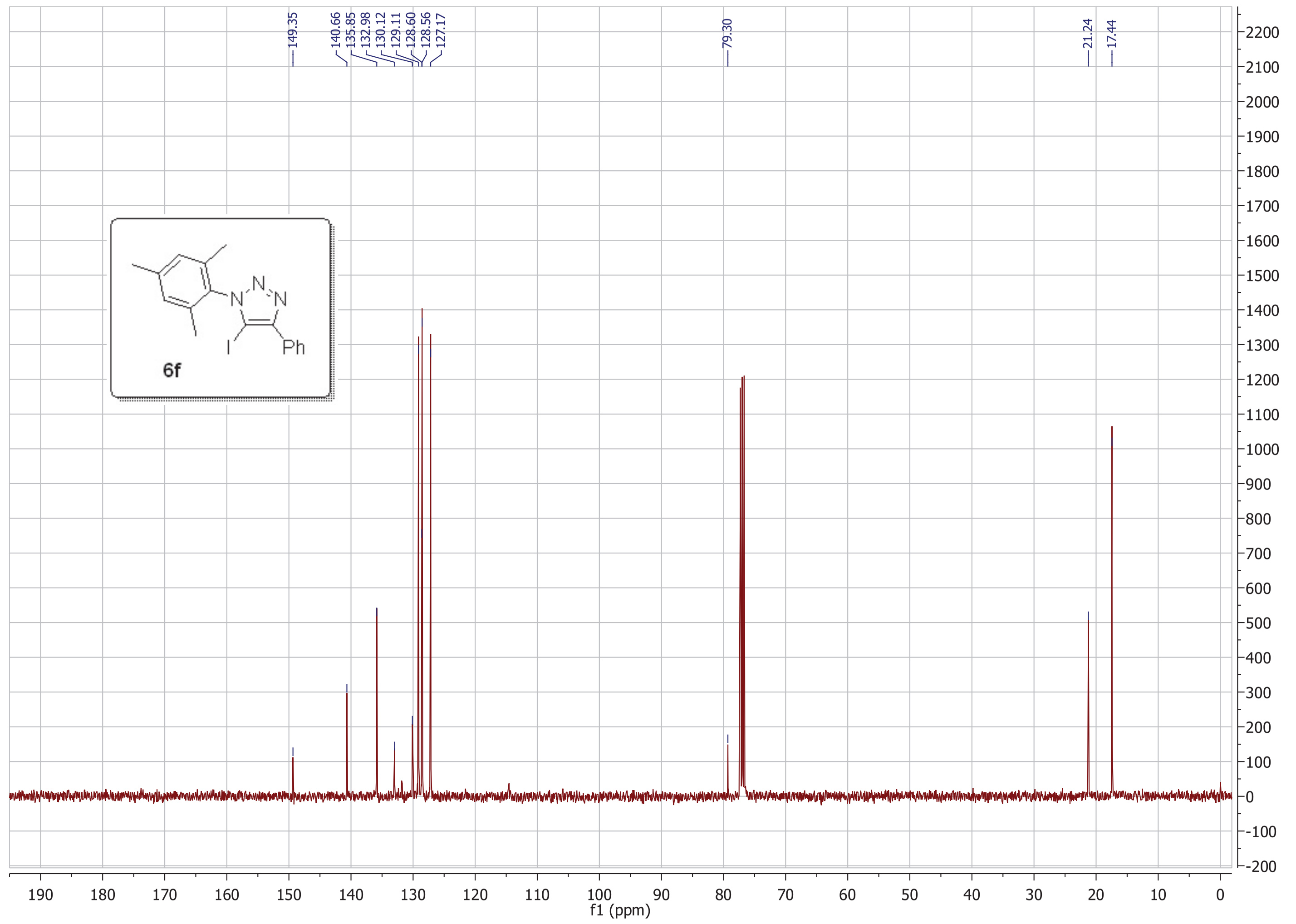
149.35
140.66
135.85
132.98
130.12
129.11
128.60
128.56
127.17
79.30
21.24
17.44
6f
Ph
I
f1 (ppm)

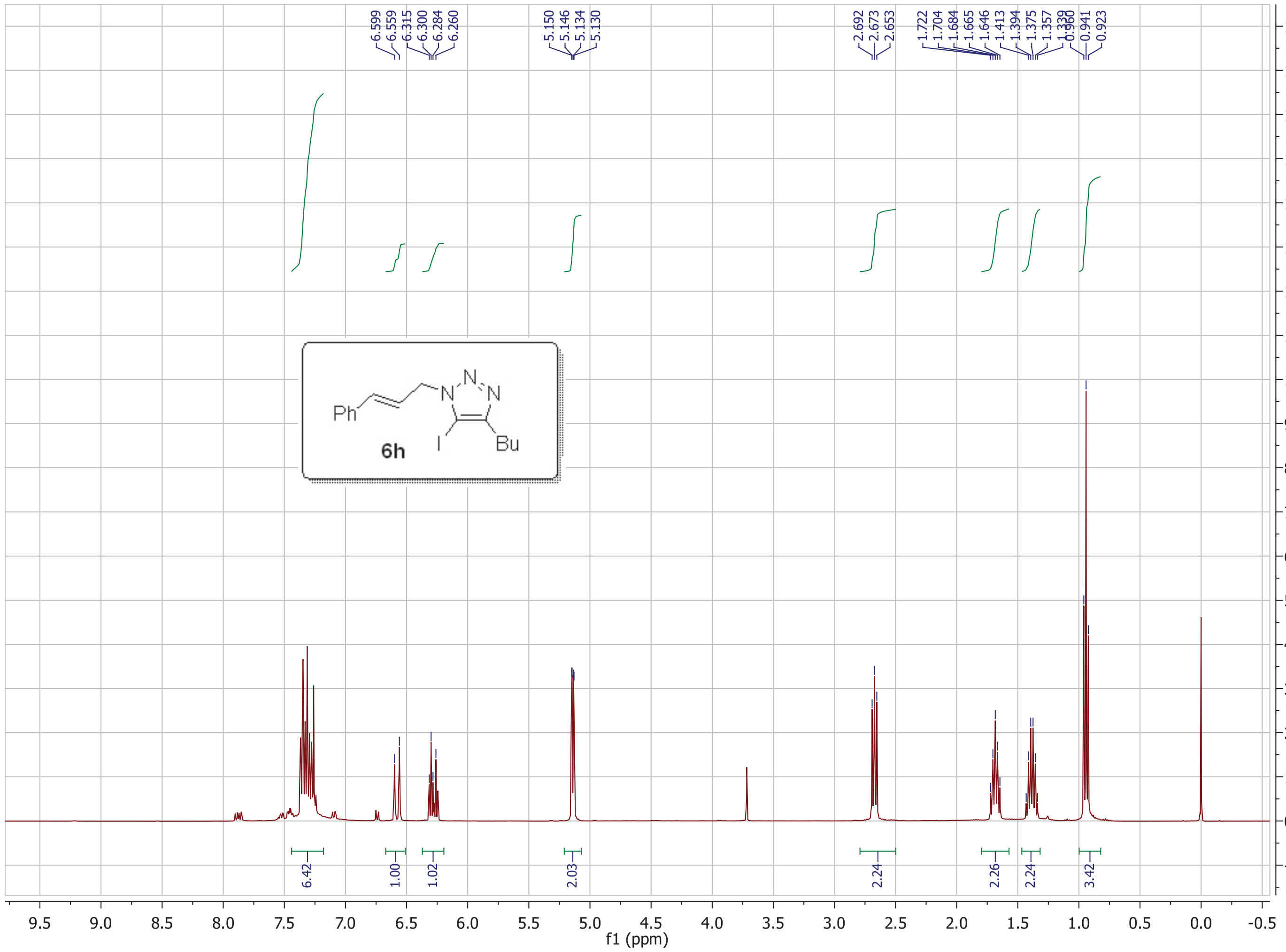
6.599
6.559
6.315
6.300
6.284
6.260
5.150
5.146
5.134
5.130
2.692
2.673
2.653
1.722
1.704
1.684
1.665
1.646
1.413
1.394
1.375
1.357
1.339
0.960
0.941
0.923
Ph
N
N
N
I
Bu
6h
6.42
1.00
1.02
2.03
2.24
2.26
2.24
3.42
f1 (ppm)

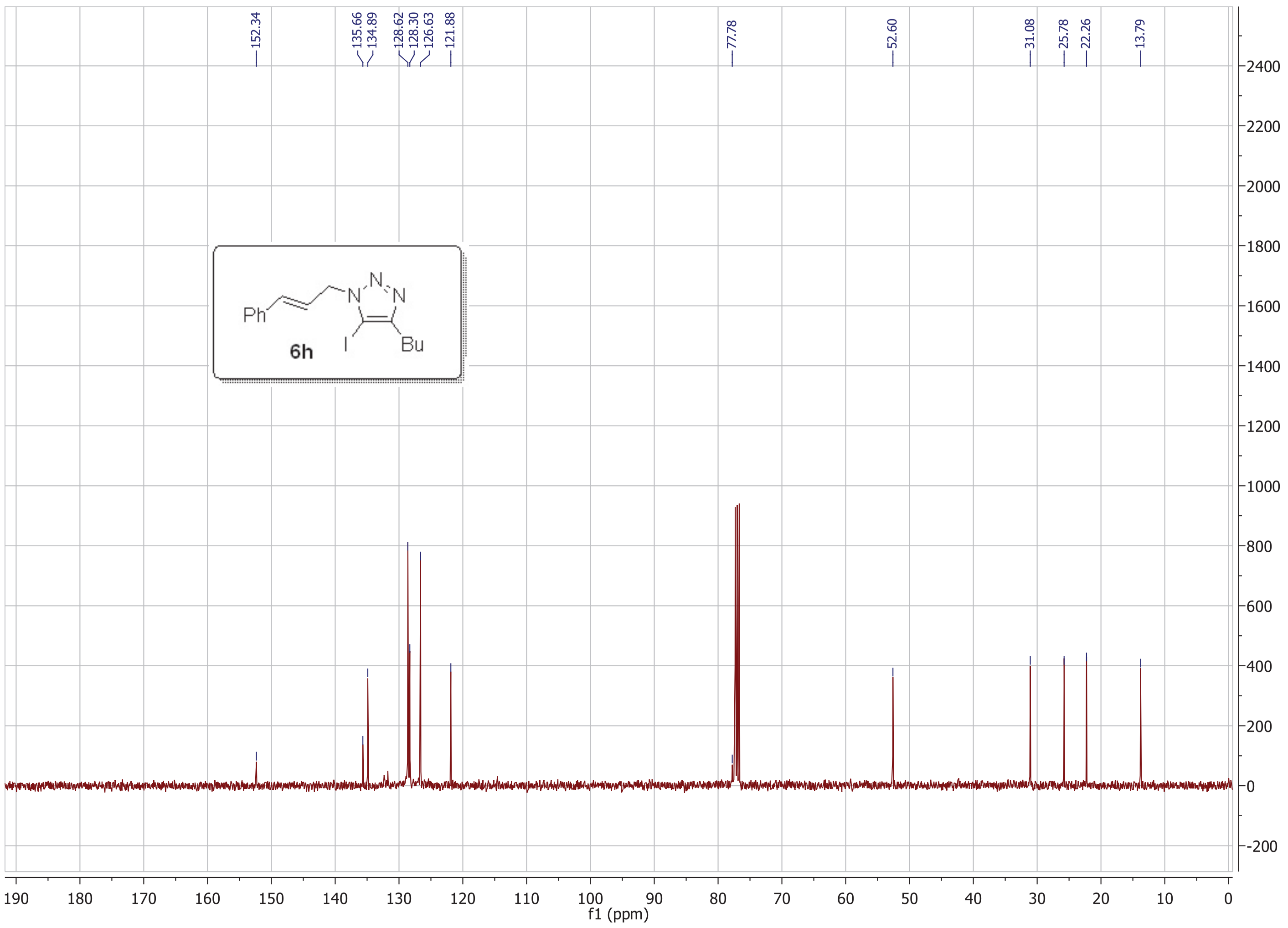
152.34
135.66
134.89
128.62
128.30
126.63
121.88
77.78
52.60
31.08
25.78
22.26
13.79
Ph
N
N
N
I
Bu
6h
f1 (ppm)

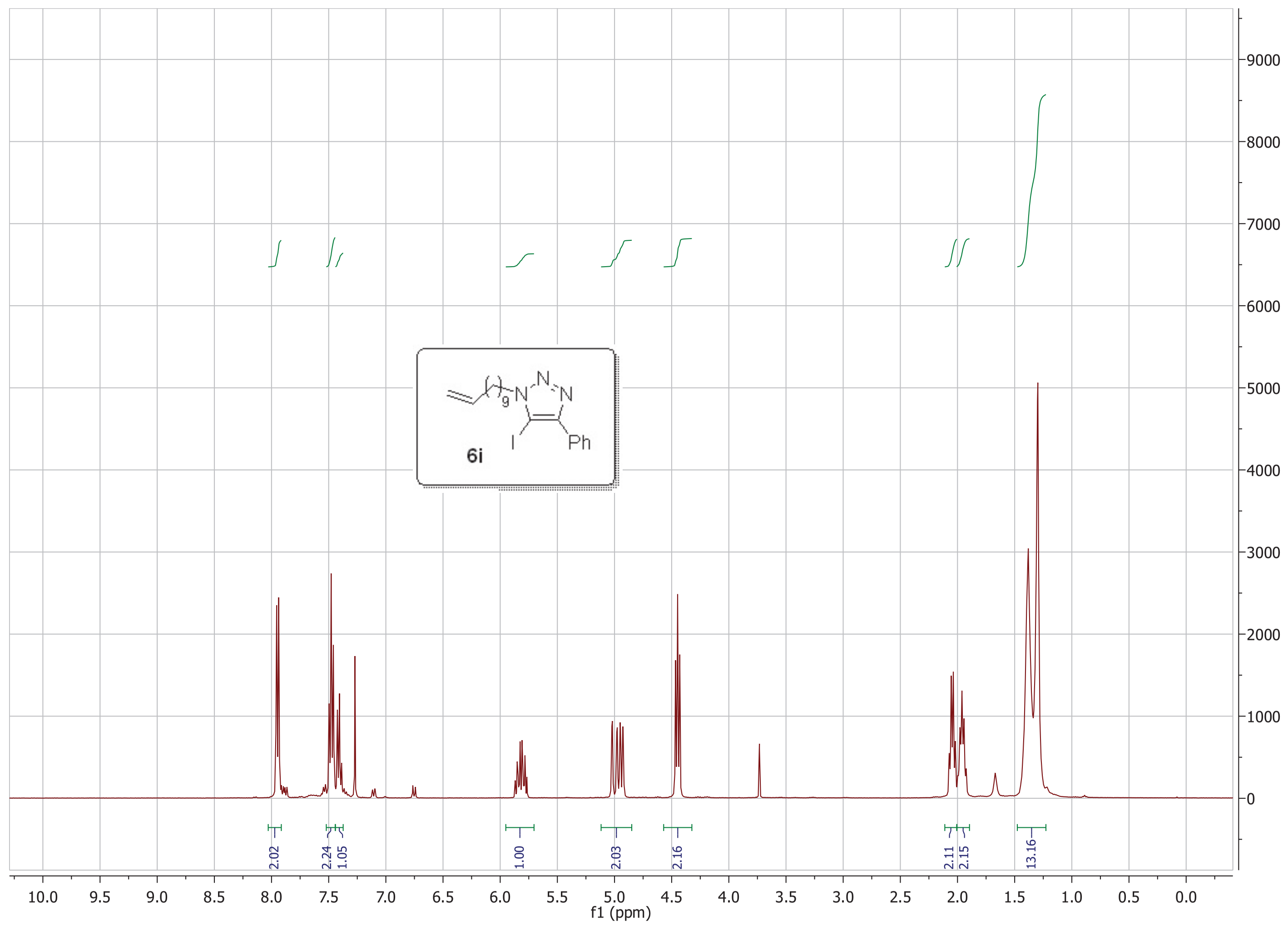
6i
Ph
I
N
9
2.02
2.24
1.05
1.00
2.03
2.16
2.11
2.15
13.16
f1 (ppm)

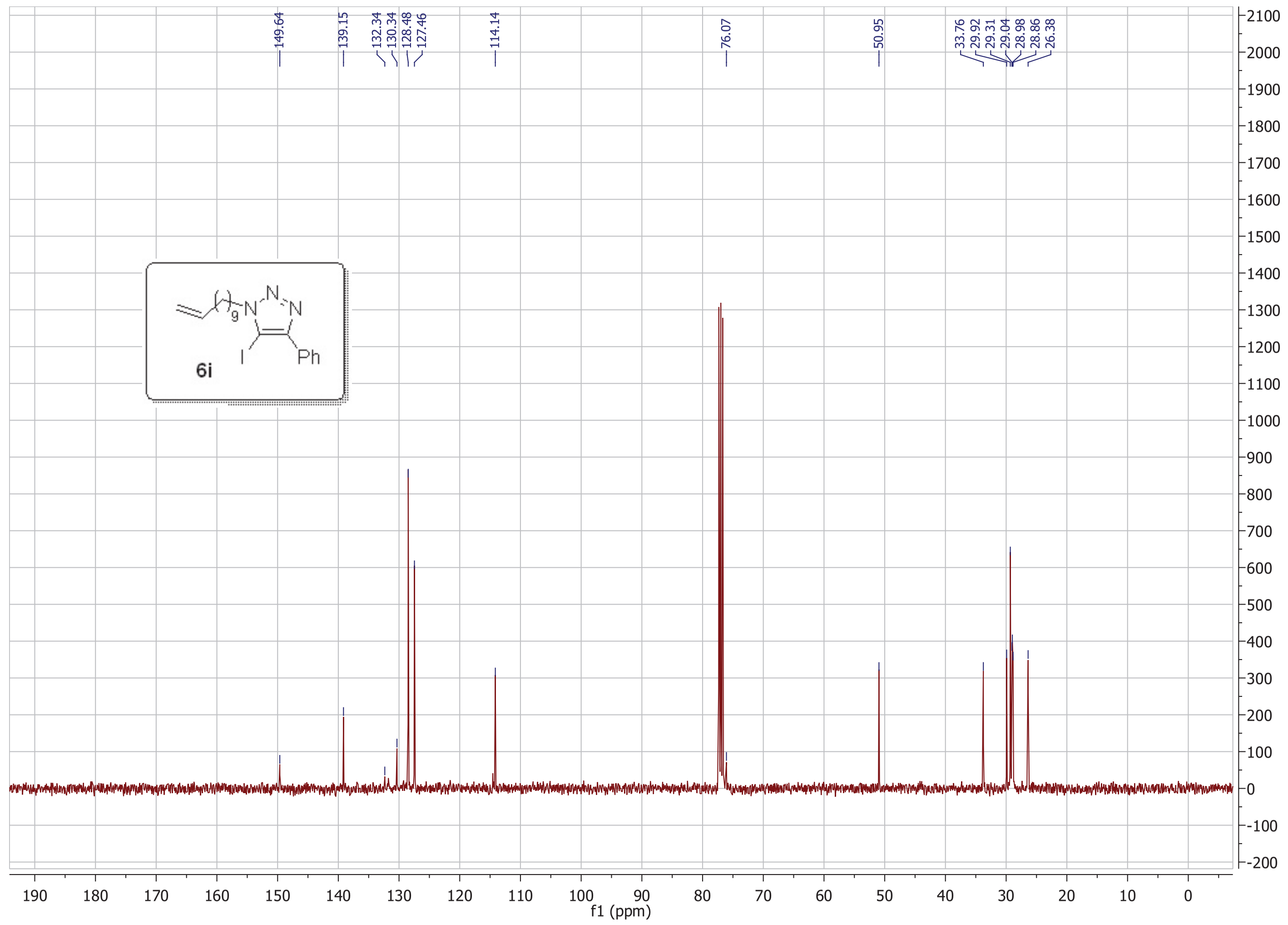
149.64
139.15
132.34
130.34
128.48
127.46
114.14
76.07
50.95
33.76
29.92
29.31
29.04
28.98
28.86
26.38
6i
Ph
I
N
9
f1 (ppm)

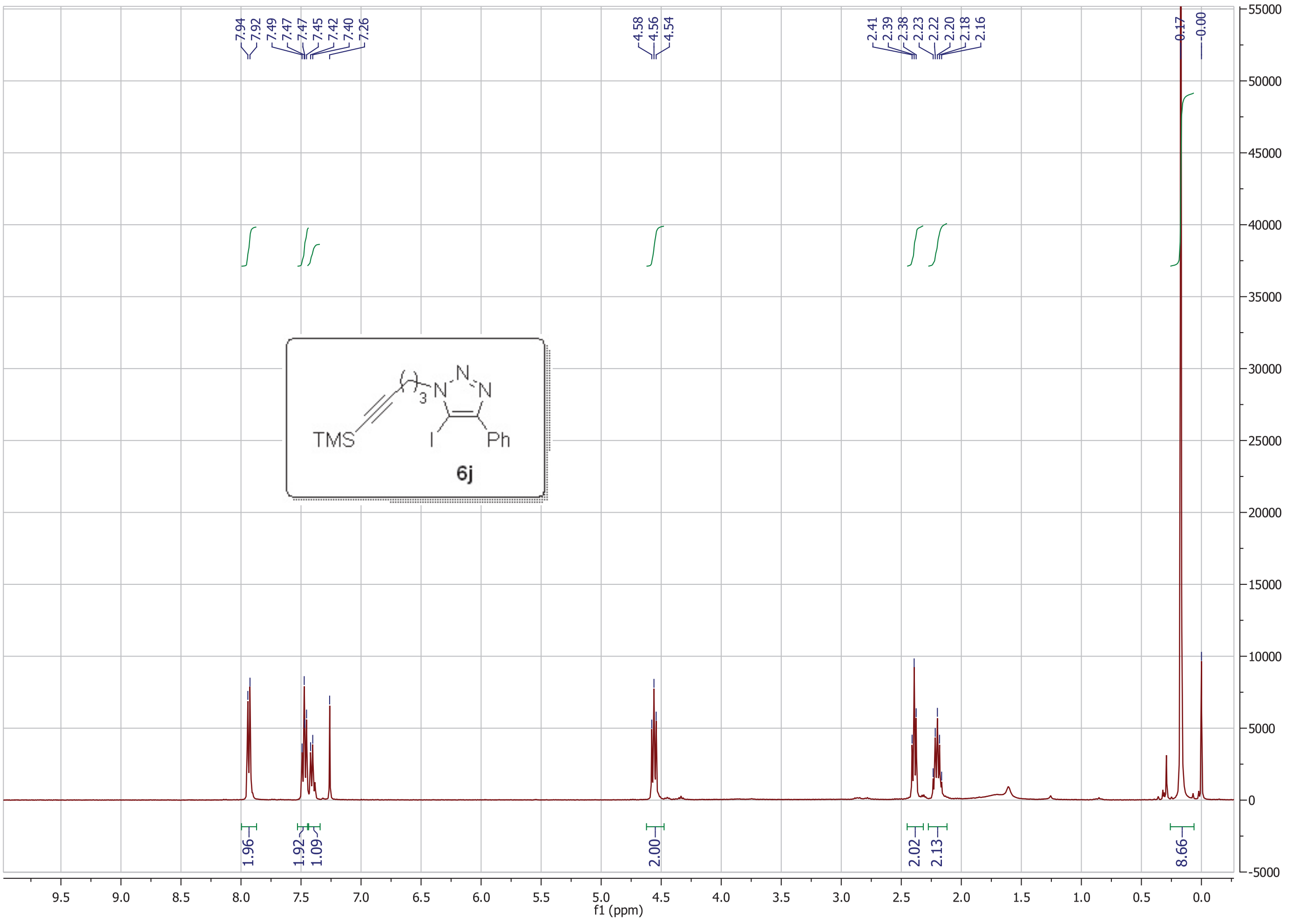
7.94
7.92
7.49
7.47
7.47
7.45
7.42
7.40
7.26
4.58
4.56
4.54
2.41
2.39
2.38
2.23
2.22
2.20
2.18
2.16
0.17
-0.00
1.96
1.92
1.09
2.00
2.02
2.13
8.66
TMS
N
N
N
3
I
Ph
6j
f1 (ppm)

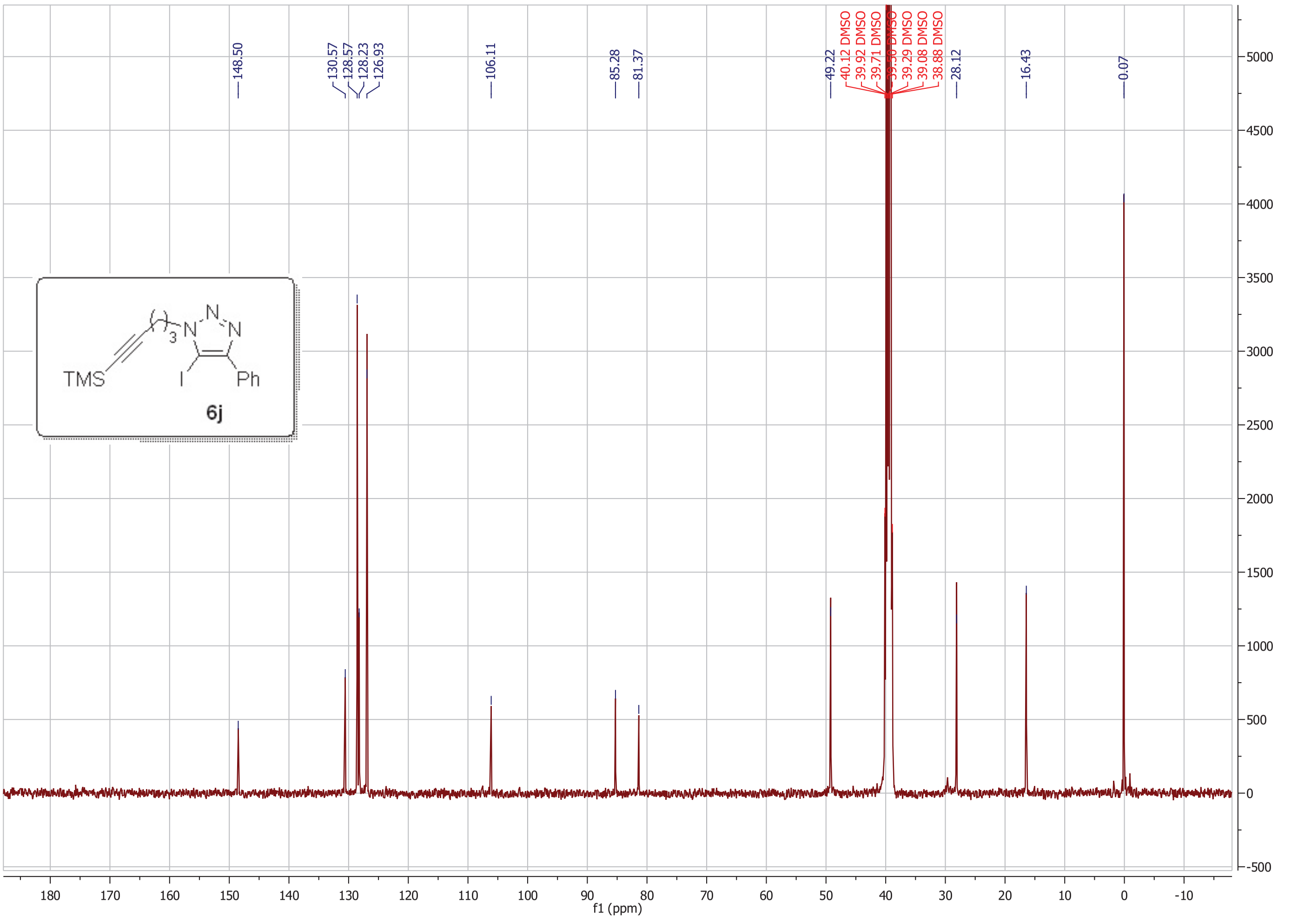
148.50
130.57
128.57
128.23
126.93
106.11
85.28
81.37
49.22
40.12 DMSO
39.92 DMSO
39.71 DMSO
39.50 DMSO
39.29 DMSO
39.08 DMSO
38.88 DMSO
28.12
16.43
0.07
TMS
N
N
N
3
I
Ph
6j
f1 (ppm)
